# Prolonged deprivation of arginine or leucine induces PI3K/Akt-dependent reactivation of mTORC1

**DOI:** 10.1016/j.jbc.2022.102030

**Published:** 2022-05-13

**Authors:** Gwen R. Buel, Huy Q. Dang, John M. Asara, John Blenis, Anders P. Mutvei

**Affiliations:** 1 Programs in Biological and Biomedical Sciences, Harvard Medical School, Boston, Massachusetts, USA; 2 Meyer Cancer Center and Department of Pharmacology, Weill Cornell Medical College, New York, New York, USA; 3 Department of Cell Biology, Harvard Medical School, Boston, Massachusetts, USA; 4 Department of Immunology, Pathology and Genetics, Uppsala University, Uppsala, Sweden; 5 Department of Medicine, Beth Israel Deaconess Medical Center and Harvard Medical School, Boston, Massachusetts, USA

**Keywords:** mTORC1, amino acid, signaling, Akt, starvation, FBS, fetal bovine serum, MEF, mouse embryonic fibroblast, mTORC1, mechanistic target of rapamycin complex 1, TBST, Tris-buffered saline with tween, TSC, tuberous sclerosis complex

## Abstract

The mechanistic target of rapamycin complex 1 (mTORC1) is a serine/threonine kinase complex that promotes anabolic processes including protein, lipid, and nucleotide synthesis, while suppressing catabolic processes such as macroautophagy. mTORC1 activity is regulated by growth factors and amino acids, which signal through distinct but integrated molecular pathways: growth factors largely signal through the PI3K/Akt-dependent pathway, whereas the availabilities of amino acids leucine and arginine are communicated to mTORC1 by the Rag-GTPase pathway. While it is relatively well described how acute changes in leucine and arginine levels affect mTORC1 signaling, the effects of prolonged amino acid deprivation remain less well understood. Here, we demonstrate that prolonged deprivation of arginine and/or leucine leads to reactivation of mTORC1 activity, which reaches activation levels similar to those observed in nutrient-rich conditions. Surprisingly, we find that this reactivation is independent of the regeneration of amino acids by canonical autophagy or proteasomal degradation but is dependent on PI3K/Akt signaling. Together, our data identify a novel crosstalk between the amino acid and PI3K/Akt signaling pathways upstream of mTORC1. These observations extend our understanding of the role of mTORC1 in growth-related diseases and indicate that dietary intervention by removal of leucine and/or arginine may be an ineffective therapeutic approach.

In order for cells to maintain homeostasis in an ever-changing environment, cells need to carefully balance anabolic reactions with catabolic reactions. This is largely orchestrated by the mechanistic target of rapamycin complex 1 (mTORC1), a highly evolutionarily conserved serine/threonine kinase complex that promotes growth-favoring processes, including protein translation, lipid synthesis, and nucleotide biosynthesis, under conditions of nutrient sufficiency (1, 2, 3, 4, 5). Among the upstream cues that regulate mTORC1 activity, growth factors and amino acids have long been known to be crucial for mTORC1 activity (6, 7). Growth factor stimulation leads to activation of PI3K/Akt signaling, which inactivates the tuberous sclerosis complex (TSC) component TSC2, leading to activation of the small GTPase Rheb, a key regulator of mTORC1 (8, 9, 10). In contrast, amino acids have been shown to communicate through a TSC2/Rheb-independent mechanism, reliant on the family of Rag small GTPases, which exists as heterodimers of RagA or RagB and RagC or RagD (2, 11, 12). When the Rag-GTPases are active, they recruit mTORC1 to lysosomes, where association with Rheb leads to mTORC1 activation when growth factors are present (13, 14, 15). Among the 20 proteinogenic amino acids, arginine and leucine are known to be key regulators of mTORC1 activity, as deprivation of these amino acids leads to rapid inhibition of mTORC1 activity (16). Recently, it was shown that cytosolic leucine and arginine are communicated to the Rag-GTPases by the Sestrin and Castor proteins, respectively, through the Gator1 and Gator2 complexes (2, 3).

While these recent advancements have increased our understanding of how mTORC1 activity is regulated during acute changes in leucine or arginine levels (2, 13, 15, 16), the effects of prolonged amino acid deprivation remain less characterized. Curiously, a few studies have observed what appears to be a reactivation of mTORC1 activity upon prolonged deprivation of individual amino acids, including histidine, glutamine, and leucine (17, 18, 19, 20), but the molecular details and extent of this reactivation remain poorly understood. Since amino acid levels may drop in an organism for more extended periods of time (21), we chose to address how prolonged arginine or leucine deprivation affects mTORC1 activity (prolonged starvation is defined in this work as 1.5–24 h and acute starvation less than an hour). Here, we demonstrate that acute leucine or arginine deprivation leads to rapid inactivation of mTORC1 but that prolonged starvation leads to potent reactivation of mTORC1 activity. Importantly, we find that this reactivation is independent of regeneration of amino acids by canonical macroautophagy, the lysosome, or the proteasome. Instead, the reactivation is accompanied by increased Akt signaling, which is necessary for the reactivation of mTORC1 activity to occur. Our results suggest that a crosstalk exists between the growth factor and amino acid regulatory arms of mTORC1 and indicate that PI3K/Akt-dependent signaling may partially compensate for a lack in amino acid signaling.

## Results

### Prolonged deprivation of leucine and arginine leads to reactivation of mTORC1 in multiple cell lines

**Figure 1:**
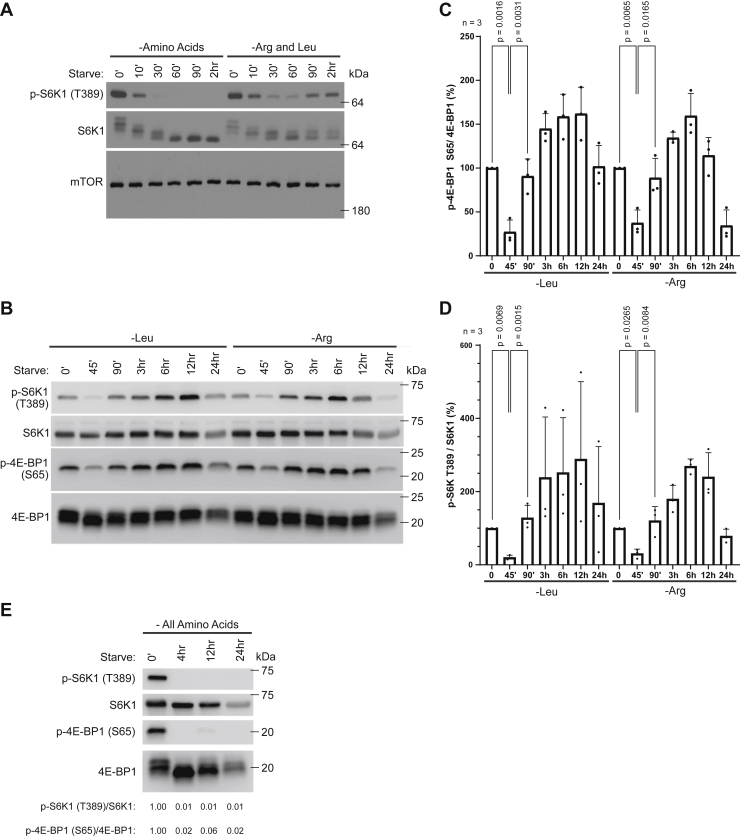
**mTORC1 reactivation follows prolonged deprivation of leucine or arginine.***A*–*E*, mTORC1-associated signaling was assessed in MEFs that had been deprived of either all amino acids (*A* and *E*), arginine (Arg) and leucine (Leu) (*A*), or arginine or leucine alone (*B*), for the indicated times, by immunoblotting endogenous proteins, as specified. Quantifications of (*B*) are shown in (*C* and *D*): graphs represent relative immunoblot band intensity from three individual experiments (n = 3). Statistical data are presented as mean values  ±  SD, one-way ANOVA with Tukey’s post hoc test. p-S6K T389/S6K1 immunoblot band intensity ratios are shown in (*E*). Experiments were repeated at least three times with equivalent results. MEF, mouse embryonic fibroblast; mTORC1, mechanistic target of rapamycin complex 1.

mTORC1 activation requires leucine and arginine (16), but how prolonged deprivation of these amino acids affects mTORC1 activity remains less characterized. To address this, we deprived mouse embryonic fibroblasts (MEFs) of either leucine and arginine, or all amino acids, and assessed the level of mTORC1 activity at multiple time points ranging from 10 min to over 90 min of starvation, by analyzing the phosphorylation status of the mTORC1 substrates S6 kinase 1 (S6K1) T389 and eIF4E-binding protein 1 (4E-BP1) S65 (6). Both starvation conditions caused a rapid suppression of mTORC1 activity (Fig. 1*A*) in agreement with previous studies (16). However, whereas mTORC1 remained inactivated in cells starved of all amino acids, a striking reactivation was observed after 90 min in leucine- and arginine-deprived cells, reaching mTORC1 signaling levels similar to those observed under amino acid sufficient conditions (Fig. 1*A*). We were curious whether this reactivation was specifically caused by deprivation of either arginine or leucine and therefore starved cells of either of these two amino acids, whereafter the phosphorylation status of mTORC1 targets S6K1 T389 and 4E-BP1 S65 were assessed at multiple time points between 45 min and 24 h post starvation. We found that the kinetics of mTORC1 inactivation and reactivation was similar between arginine- and leucine-deprived cells (Figs. 1, *B*–*D* and S1*A*), indicating that the response is not unique to one of the amino acids. Interestingly, reactivated mTORC1 remained at levels comparable to that found during amino acid–sufficient levels for up to 12 h post leucine or arginine deprivation (Fig. 1, *B*–*D*). An equivalent mTORC1 reactivation was observed when assessing the phosphorylation status of mTORC1 targets ULK1 S757 and Grb10 S476, although the kinetics and magnitude of Grb10 S476 were different between leucine and arginine starvation (Fig. S1*B*). In contrast, mTORC1 reactivation was not observed during a 24 h time course of total amino acid deprivation (Figs 1*E* and S1, *C* and *D*).

**Figure 2:**
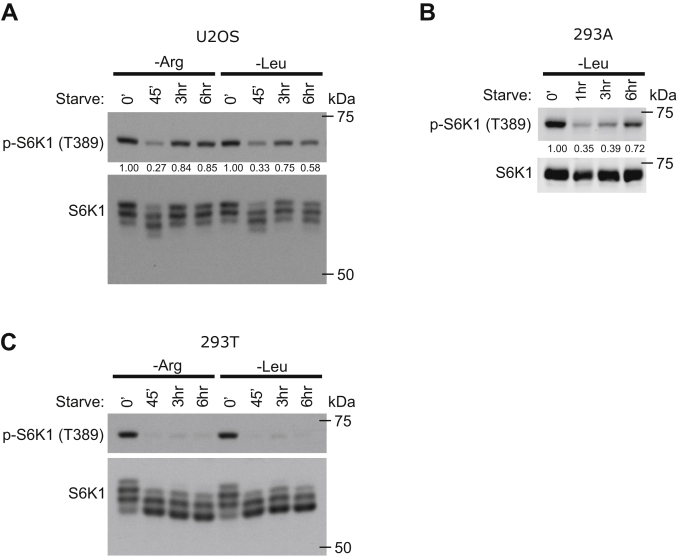
**Analysis of mTORC1 reactivation in additional cell lines.***A*–*C*, U2OS cells (*A*), 293A cells (*B*) or 293T cells (*C*) were deprived of arginine or leucine for the indicated times and analyzed by immunoblotting for the indicated proteins. p-S6K T389/S6K1 immunoblot band intensity ratios are shown. Experiments were repeated at least twice with equivalent results. mTORC1, mechanistic target of rapamycin complex 1.

We next explored the mTORC1 reactivation response in three additional cell lines: the osteosarcoma cell line U2OS and the human embryonic kidney cell lines 293A and 293T cells. In both U2OS and 293A cells, prolonged arginine and leucine deprivation led to a clear reactivation of mTORC1, comparable to our results obtained in MEFs (Fig. 2, *A* and *B*). However, in 293T cells, where the mTORC1 pathway is not strongly regulated by serum or insulin (22), reactivation was negligible (Fig. 2*C*), indicating that the mTORC1 reactivation is not observed in all cell lines. Taken together, our data show that prolonged leucine or arginine deprivation leads to reactivation of mTORC1 activity in multiple cell types.

### The mTORC1 reactivation occurs without the regeneration of amino acids from lysosomal, proteasomal, or canonical autophagy-mediated protein degradation

**Figure 3:**
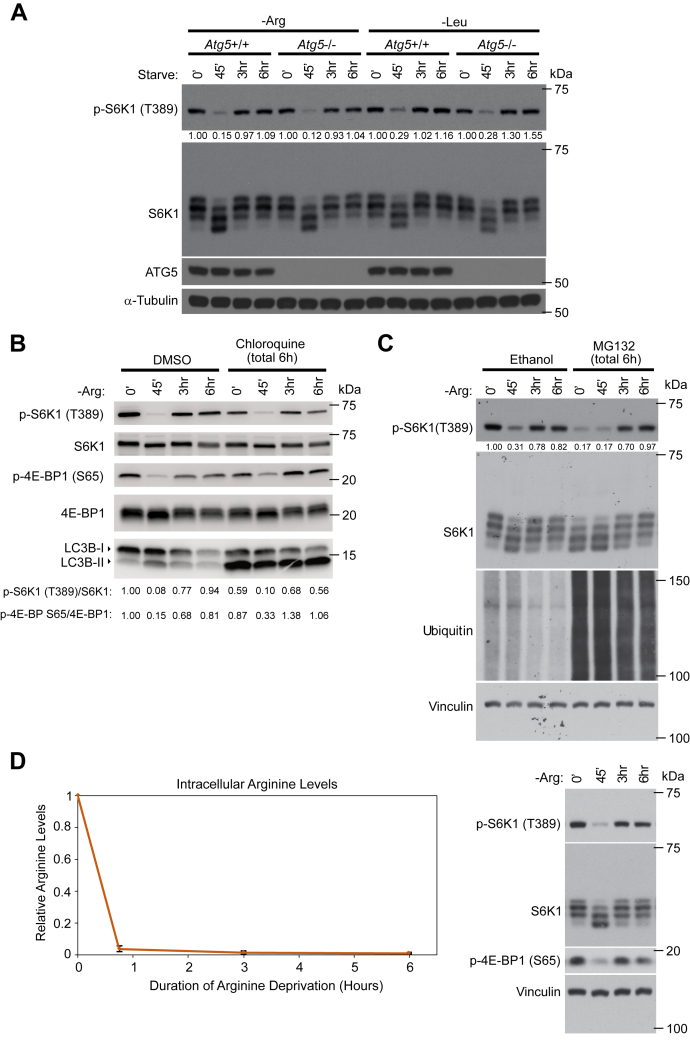
**mTORC1 reactivation is independent of canonical autophagy or proteasomal degradation of proteins.***A*, autophagy deficient *Atg5*^-/-^ MEFs or WT littermate controls were deprived of arginine or leucine for the indicated times. *B* and *C*, MEFs were deprived of arginine in the presence of 100 μM of the lysosomal inhibitor chloroquine (*B*) or 20 μM of the proteasomal inhibitor MG132 (*C*) as indicated. All drug treatments in (*B*) and (*C*) were for a total of 6 h *D*, MEFs were deprived of arginine for 0 min, 45 min, 3 h, or 6 h, and intracellular metabolite levels were analyzed *via* mass spectrometry. The graph on the left shows arginine levels with SDs averaged from four separate experiments (*left panel*). mTORC1 associated signaling was assessed in duplicate plates by immunoblotting the specified endogenous proteins (*right*). Experiments were repeated at least three times. p-S6K T389/S6K1 (*A*–*C*) and p-4E-BP1 S65/4E-BP1 (*B*) immunoblot band intensity ratios are shown. MEF, mouse embryonic fibroblast; mTORC1, mechanistic target of rapamycin complex 1.

When nutrients are limited, cells adapt by inducing macroautophagy (hereafter referred to as autophagy) to degrade proteins and other cytosolic macromolecules (23). The release of amino acids *via* canonical autophagy has been proposed to cause mTORC1 reactivation under certain conditions (18), but it remains unclear whether mTORC1 reactivation can also occur in a manner that is independent of autophagy. To explore this, we tested whether mTORC1 was reactivated in autophagy-deficient MEFs that lack *Atg5*, which is essential for canonical autophagosome biogenesis (24). Intriguingly, a robust mTORC1 reactivation was also observed in autophagy-deficient *Atg5*^*-/-*^ MEFs during prolonged arginine or leucine deprivation (Fig. 3*A*), demonstrating that this response can occur independently of canonical autophagy. To further investigate if the reactivation response was driven by a regeneration of amino acids through either lysosomal or proteasomal degradation of proteins, we treated cells with the lysosomal degradative inhibitor chloroquine, or the proteasomal inhibitor MG132, for 6 h in combination with arginine deprivation. Although baseline mTORC1 was affected by these inhibitors, neither inhibition of the lysosome or the proteasome prevented the reactivation (Fig. 3, *B* and *C*), suggesting that mTORC1 reactivation can occur without the regeneration of amino acids through these protein degradation machineries. Further arguing against the notion that regeneration of arginine was driving the reactivation of mTORC1, no increase in intracellular arginine levels was observed during the time course of mTORC1 reactivation, as measured by LC/MS (Fig. 3*D*). In sum, these data suggest that the mTORC1 reactivation response can occur without the regeneration of amino acids.

### Inhibition of PI3K or Akt signaling blocks the mTORC1-reactivation response

**Figure 4:**
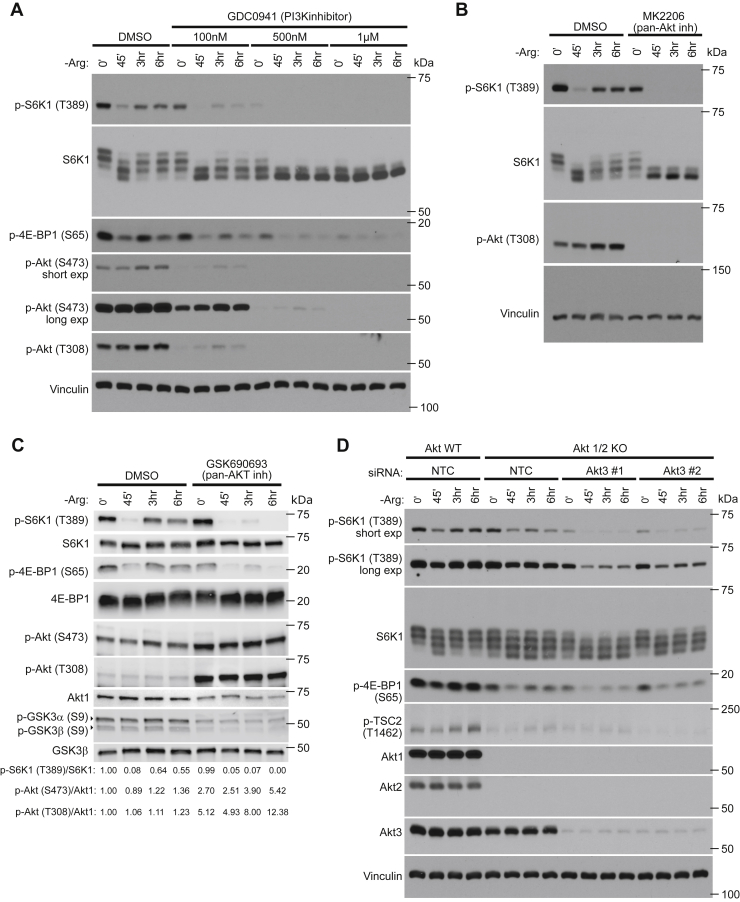
**mTORC1 reactivation requires PI3K/Akt activity.***A*, MEFs treated with the PI3K inhibitor GDC0941 at the indicated concentrations in combination with deprivation of arginine for the indicated times. Cells were treated with the PI3K inhibitor for a total of 6 h in all conditions. *B* and *C*, MEFs were deprived of arginine for the indicated time, while being treated with 2 μM MK2206 (pan-Akt inhibitor) (*B*) or 10 μM GSK690693 (pan-Akt inhibitor) (*C*) for a total of 6 h. *D*, mTORC1 and Akt signaling were analyzed after silencing of Akt3 by two different siRNAs in Akt1-2 double KO MEFs that had been deprived of arginine for the indicated times. Experiments were repeated at least twice with equivalent results. p-S6K T389/S6K1, p-Akt S473/Akt1, and p-Akt T308/Akt1 immunoblot band intensity ratios are shown in (*C*). MEF, mouse embryonic fibroblast; mTORC1, mechanistic target of rapamycin complex 1.

Given that mTORC1 reactivation was found to be independent of regeneration of amino acids, we interrogated additional signaling components that are involved in mTORC1 activation, to explore their role in the mTORC1-reactivation response. In addition to amino acids, mTORC1 activation is mediated by growth factor signaling through the PI3K–Akt–TSC2 axis (6). Interestingly, when PI3K signaling was pharmacologically inhibited by GDC0941 during prolonged arginine deprivation, mTORC1 reactivation was severely blunted (Fig. 4*A*), suggesting that the PI3K pathway is needed for reactivation to occur. To investigate whether Akt signaling was also required for mTORC1 reactivation, we treated cells with two inhibitors of Akt; the PH-domain inhibitor MK2206 (Fig. 4*B*) or the ATP competitive inhibitor GSK690693 (Fig. 4*C*). As with PI3K inhibition, pharmacological inhibition of Akt significantly blunted mTORC1 reactivation (Fig. 4, *B* and *C*), again suggesting a role for PI3K/Akt signaling in the mTORC1 reactivation response. To further validate this, we silenced Akt3 in Akt1/Akt2 double KO MEFs using siRNA, to generate cells nearly completely deficient in all three isoforms of Akt, and deprived these cells of arginine. Indeed, Akt-deficient cells mirrored pharmacological inhibition of PI3K/Akt signaling, as these cells also exhibited a severely blunted mTORC1 reactivation (Fig. 4*D*). Taken together, these data demonstrate that intact PI3K/Akt signaling is needed for mTORC1 reactivation to occur.

### mTORC1 reactivation correlates with an increase in Akt signaling

**Figure 5:**
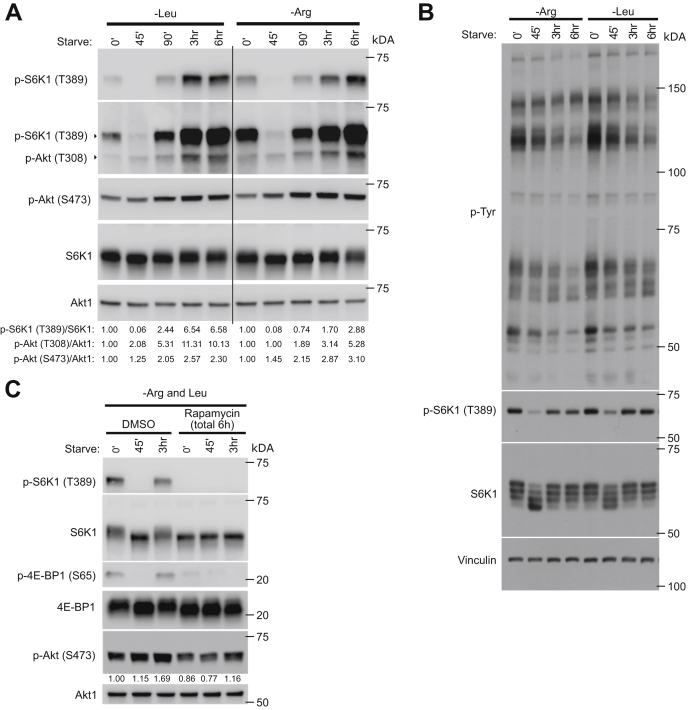
**Prolonged arginine or leucine deprivation leads to increased Akt signaling.***A*–*C*, mTORC1, Akt and growth factor–associated signaling was assessed in MEFs deprived of arginine or leucine (*A* and *B*) or arginine and leucine (*C*) for the indicated times, by immunoblotting endogenous proteins, as indicated. *A*, one of the membranes was first immunoblotted for p-S6K T389 and then reprobed for p-Akt T308, as indicated. *C*, cells were treated with rapamycin for a total of 6 h, or DMSO as a control, and starved for the indicated time. Experiments were repeated three time except for (*C*), which was repeated twice. p-S6K T389/S6K1 (*A*), p-Akt S473/Akt1 (*A* and *C*), and p-Akt T308/Akt1 (*A*) immunoblot band intensity ratios are shown. DMSO, dimethyl sufoxide; MEF, mouse embryonic fibroblast; mTORC1, mechanistic target of rapamycin complex 1.

Since blockage of PI3K/Akt signaling inhibited mTORC1 reactivation, we next determined Akt activation levels during the time course of mTORC1 reactivation by assessing the phosphorylation state of Akt at residues T308 and S473 (25). Interestingly, Akt activation was increased upon prolonged arginine or leucine deprivation in MEFs (Fig. 5*A*), U2OS cells (Fig. S2*A*) and 293A (Fig. S3*D*), indicating that prolonged deprivation of arginine or leucine increases Akt signaling. However, Akt signaling was not increased in 293T cells (Fig. S2*B*), where we did not observe any reactivation of mTORC1 (Fig. 2*C*). To investigate if the increase in Akt signaling was coupled to a global increase in growth factor signaling in cells, we probed lysates that had been starved of arginine or leucine with an antiphosphotyrosine antibody, since the majority of growth factor signaling is mediated through tyrosine phosphorylation (26). Interestingly, we did not find any evidence of a general increase in growth factor signaling activity, as overall phosphotyrosine levels instead robustly decreased during the arginine or leucine deprivation time course (Fig. 5*B*), suggesting that arginine and leucine deprivation have a more specific effect on Akt signaling.

Inhibition of mTORC1 induces Akt signaling through a series of feedback loops (27, 28, 29, 30). We therefore investigated whether the increase in Akt signaling was driven by loss of mTORC1 activity by arginine or leucine starvation. To this end, Akt S473 phosphorylation was assessed in cells where mTORC1 signaling had been inhibited for 3 h with rapamycin prior to a 3 h time course of leucine and arginine deprivation, during which cells were still treated with rapamycin. mTORC1 inhibition did not further increase Akt signaling during the starvation course (Fig. 5*C*), suggesting that the loss of mTORC1 activity is not a major driver of this response. We next considered whether the reactivation response was driven by a decline of intracellular glutamine levels, which is known to increase mTORC2-mediated Akt signaling (31). However, LC/MS analysis revealed no significant changes in intracellular glutamine levels during leucine or arginine starvation (Fig. S3*A*). Moreover, treating arginine-deprived cells with excess glutamine (8 mM instead of 4 mM L-glutamine) for a total of 6 h during the course of arginine starvation did not suppress mTORC1 or Akt signaling, which instead increased at the 0′ and 45′ starvation time points (Fig. S3*B*), suggesting that a decrease in intracellular glutamine level is not driving the mTORC1-reactivation response.

**Figure 6:**
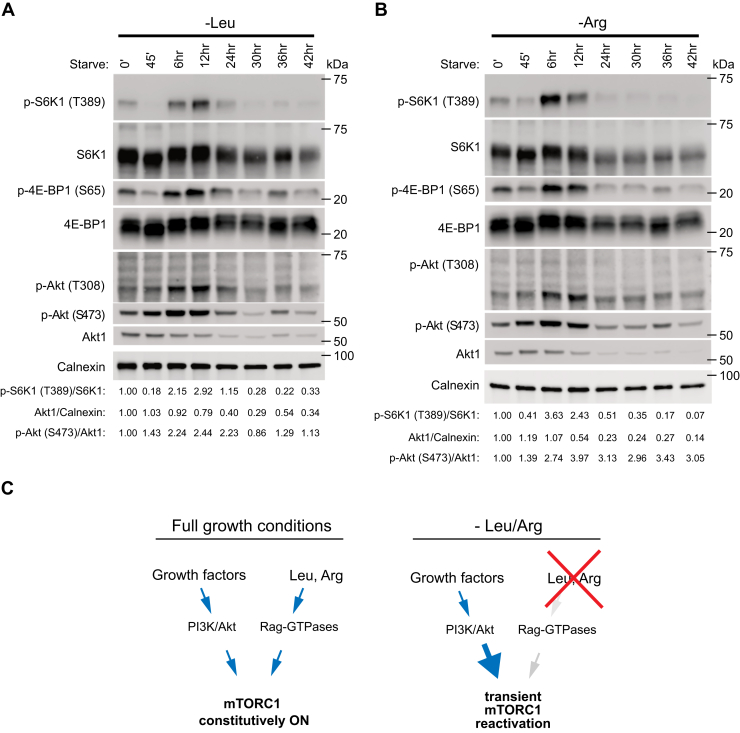
**The increase in Akt signaling upon prolonged leucine or arginine deprivation is transient.***A* and *B*, mTORC1 and Akt associated signaling was assessed in MEFs that had been deprived of leucine (*A*) or arginine (*B*) for the indicated times. The experiment was repeated at least three times. p-S6K T389/S6K1, Akt/Calnexin, p-Akt S473/Akt1, and p-Akt T308/Akt1 immunoblot band intensity ratios are shown in (*A*) and (*B*). *C*, graphical summary of findings. In cells that are growing and proliferating, mTORC1 is constitutively activated by growth factor PI3K/Akt signals and amino acid Rag-GTPase signals, communicated to mTORC1 through distinct upstream molecular pathways (*left*). Prolonged deprivation of arginine or leucine leads to loss of Rag-GTPase signaling, a potent increase in PI3K/Akt signaling and a transient reactivation of mTORC1. The reactivation of mTORC1 is dependent on PI3K/Akt (*right*). MEF, mouse embryonic fibroblast; mTORC1, mechanistic target of rapamycin complex 1.

Since arginine and leucine signal to mTORC1 through the Rag-GTPases, we next investigated whether the reactivation response was dependent on this pathway. Indeed, mTORC1 remained active in Rag A/B-deficient cells subjected to leucine or arginine starvation (Fig. S3*C*), consistent with the important role of this pathway in leucine and arginine signaling to mTORC1 (2, 32). Interestingly, Rag A/B-deficient cells also displayed elevated Akt S473 phosphorylation compared to control cells (Fig. S3*D*), in line with previous observations (33). Together, these results indicate that suppression of amino acid signaling may also lead to upregulation of Akt signaling in other cellular contexts. To learn whether Akt signaling and reactivated mTORC1 signaling correlated over longer periods of arginine and leucine deprivation, we determined the levels of both total Akt and phosphorylated Akt at S473 and T308 for up to 42 h of starvation. Both total and phosphorylated Akt started to decline after 6 to 12 h of starvation (Fig. 6, *A* and *B*). Notably, this drop in Akt levels and phosphorylation after 12 h of starvation correlated with a decrease in mTORC1 signaling (Fig. 6, *A* and *B*). Taken together, our data suggest that sustained arginine or leucine deprivation leads to a specific upregulation of Akt signaling through an unknown mechanism, which is involved in mTORC1 reactivation (Fig. 6*C*).

## Discussion

mTORC1 integrates a panoply of intracellular and extracellular cues, including amino acid signals and growth factor signals, to direct cellular growth (5). Previous studies have established that growth factors and amino acids are communicated to mTORC1 through distinct molecular pathways (10, 13, 34). Growth factors activate mTORC1 through the PI3K/Akt-dependent pathway, whereas leucine and arginine, the key amino acids in mTORC1 regulation (16), activate mTORC1 through the Rag-GTPase pathway (2, 13, 35, 36). However, these studies have mostly focused on acute amino acid starvation, during which mTORC1 is rapidly inactivated, and as a result, the effects of prolonged amino acid deprivation remain less understood.

We find that, contrary to acute starvation, prolonged leucine or arginine deprivation leads to a potent mTORC1 reactivation. Although this observation is intriguing on its own, we currently do not have a clear understanding of what physiological role this mTORC1 reactivation serves. We speculate that this could be a part of an uncharacterized cellular adaptation response, which allows cells to maintain essential cellular functions during milder forms of amino acid starvation, in the expectation that levels of amino acid will be restored in the near future. These findings suggest, however, that therapies targeting the leucine or arginine signaling pathways (37, 38) may underperform due to reactivation of the mTORC1 pathway.

Prolonged deprivation of amino acids histidine, leucine, and glutamine has previously been shown to lead to reactivation of mTORC1 (17, 18, 19, 20, 31). We now extend these observations also to prolonged arginine deprivation. Moreover, it has been reported that mTORC1 reactivation occurs due to regeneration of amino acids through canonical autophagy (18). We find that mTORC1 reactivation upon prolonged arginine and leucine deprivation can occur independently of Atg5- or lysosome-mediated autophagy or proteasomal function (Fig. 3). Although we cannot rule out that amino acids are being regenerated through other less characterized forms of protein degradation, such as noncanonical modes of autophagy, our metabolic profiling analysis shows no sign of arginine being regenerated during the time course of deprivation. Together, these data suggest that mTORC1 reactivation upon prolonged arginine or leucine deprivation can occur independently of the regeneration of these amino acids.

Importantly, our data demonstrate that sustained deprivation of arginine or leucine leads to an increase in Akt signaling, which is essential for the mTORC1 reactivation to occur. Although, we have not identified the specific mechanisms that elevate the growth factor signaling arm, we find that the response is not driven solely by mTORC1 inactivation by leucine or arginine starvation, which induces PI3K/Akt signaling through several feedback loops, initiated, for example, by loss of mTORC1-dependent phosphorylation of Grb10, an inhibitor of PI3K signaling (29, 30). Altogether, our data support a model where PI3K-dependent Akt signaling may be able to transiently compensate for the lack of amino acid signals to mTORC1. In support of this notion, we also observe increased Akt signaling in RagA/B-GTPase–deficient cells, which lack the ability to signal leucine and arginine to mTORC1 (32). Prolonged glutamine or glucose starvation was previously found to increase basal Akt signaling levels by promoting mTORC2 activity (31, 39). Although, we find no evidence of that prolonged arginine or leucine deprivation decreases intracellular glutamine levels, the role of mTORC2 in this response is yet to be determined. Further work will be necessary to understand the complex interactions between the amino acid and growth factor–signaling components upstream of mTORC1 and the physiological roles that they serve.

In conclusion, we propose that prolonged arginine or leucine deprivation leads to a PI3K/Akt-dependent reactivation of mTORC1. This novel interplay between the mTORC1 amino acid and growth factor–signaling components provides a new layer of complexity to mTORC1 regulation, which may be of importance for therapeutic interventions, especially those involving specific amino acid depletions such as has been proposed for numerous cancer types (37, 38, 40, 41).

## Experimental procedures

### Cell culture and transfections

All cell lines were cultured in high glucose Dulbecco's modified Eagle's medium (DMEM), without pyruvate, supplemented with 10% fetal bovine serum (FBS) (Sigma–Aldrich) at 37 °C with 5% CO_2_. MEFs lacking p53 (p53^˗/˗^) were a kind gift from Dr David Kwiatkowski (Brigham and Women’s Hospital). *Atg5*^+/+^ and *Atg5*^-/-^ MEFs were generously provided by Dr N. Mizushima (24) The Akt1/Akt2 double KO MEFs and littermate controls were a kind gift from Dr M. Birnbaum. HEK 293A RagA and RagB CRISPR/Cas9 KO cells and HEK 293A control cells were kindly provided by Drs Jenna Jewell and Kun-Liang Guan (32). siRNAs targeted against Akt3 were purchased from Sigma–Aldrich: SASI_Mm01_00200790 (Akt3 #1) and SASI_Mm01_00200791 (Akt3 #2), and SIC001 Mission siRNA Universal Negative Control. siRNA transfections were performed with Lipofectamine RNAiMax (Life Technologies).

### Amino acid deprivations

Media used for amino acid deprivations were formulated as normal high glucose (4.5 g/L) DMEM without pyruvate, lacking the indicated amino acid/amino acids, or complete to be used as control full amino acid containing media. Media was either purchased from the Memorial Sloan Kettering Cancer Center Media Core or prepared based on the formulation provided by ThermoFisher. Amino acid–deficient or control media were supplemented with 10% dialyzed FBS (Sigma–Aldrich) to avoid inadvertently adding amino acids to the media from the FBS. To avoid cell detachment during media changes, 20 mg/ml fibronectin (Corning) in PBS was added to the dish for 1 h prior to cell plating or laminin (Sigma) at 10 μg/ml in PBS for 4 to 24 h at 37 degrees. For laminin coating, cells were first incubated with 0.01% poly-L-ornithine solution for 30 min.

For the amino acid–deprivation experiments, the cells were washed with and replaced with the appropriate media, either complete amino acid media as a control or the individual amino acid–deficient media for the time frame described in each experiment. The experimental condition indicated as “0” refers to cells cultured in amino acid complete media for the time of the longest treatment.

### Antibodies and reagents

The following antibodies were purchased from Cell Signaling Technologies: p-4E-BP1 S65 (#9451), p-S6K1 T389 (#9234), S6K1 (#2708), mTOR (#2983), p-TSC2 T1462 (#3611), p-Akt T308 (#13038), p-Akt S473 (#4060), p-ULK S757 (#6888), ULK1 (8054), 4E-BP1 (#9644), ubiquitin (#3933), ATG5 (#12994), Akt1 (#2938), Akt2 (#3063), Akt3 (#14982), pan-Akt (#4691), p-GSK3α/β S21/9 (#9331), GSK3 α/β (5676), p-GRB10 Ser476 (11817), Grb10 (3702), RagA/B (#4357), and p-Tyr (#8954). Antibodies to vinculin (V9264) and GAPDH (G8795) were purchased from Sigma. The antipuromycin antibody clone 12D10 (MABE343) was purchased from EMD-Millipore. Calnexin was purchased from Abcam (ab22595) and LC3B from Novus Biologicals (NB100-2220). Rapamycin (used at a final concentration of 50 nM) and MG132 were purchased from Calbiochem. MK2206 was purchased from Medchem express. GSK690693 and GDC0941 were purchased from Selleck Chemicals. Chloroquine was purchased from Invitrogen; puromycin and cycloheximide were purchased from Sigma–Aldrich.

### Cell lysis and Western blotting

Cell lysis was performed on ice following a wash with ice-cold PBS. The lysis buffer contained 10 mM KPO_4_, 1 mM EDTA, 5 mM EGTA, 10 mM MgCl_2_, 0.5% NP-40, 0.1% Brij-35, 0.1% deoxycholate, 1 mM sodium vanadate, 50 mM beta-glycerophosphate, 400 μM PMSF, 0.02 μg/μl leupeptin, 0.1 μg/μl pepstatin A, and 0.02 μg/μl aprotinin, or RIPA buffer with cOmplete protease inhibitors and PhosSTOP phosphatase inhibitors (Roche). Cellular lysates were either run on large SDS-PAGE gels overnight and transferred to 0.2 μm nitrocellulose membranes at 4 °C for at least 4 h or run on Any kD Mini-PROTEAN TGX Stain-Free precast gels from Bio-Rad Laboratories and transferred to nitrocellulose membranes using the semidry Trans-Blot system (Bio-Rad Laboratories). Membrane blocking was performed with 5% milk in Tris-buffered saline with tween (TBST) or EveryBlot Blocking Buffer (Bio-Rad Laboratories). Primary antibody incubations were performed overnight in TBST with 7% bovine serum albumin or EveryBlot Blocking Buffer. Several of the immunoblots, including p-S6K T389 and total S6K1 or p-4E-BP1 and total 4E-BP1, had to be processed on separate blots due to technical reasons. Secondary antibody (horseradish peroxidase from GE healthcare) incubations were performed in 5% milk in TBST at room temperature for 2 h. Membranes from Bio-Rad Laboratories were captured with an Amersham Imager 680 (GE Healthcare), using SuperSignal West Pico PLUS Chemiluminescent Substrate from Thermo Scientific. In Figure 2*B*, secondary antibodies from LI-COR Biosciences were utilized and membranes were imaged using an Odyssey CLx Infrared Imaging System (LI-COR Biosciences). Immunoblot band intensity ratios were quantified using Fiji/ImageJ (https://imagej.net).

### Metabolite profiling

Cells were starved of arginine or leucine for the indicated time points as described previously. All conditions received fresh media with fresh dialyzed FBS at each time point. Cells were then washed once with ice-cold PBS on ice, then flash frozen in liquid nitrogen, and placed on dry ice. Cells were stored at −80 °C. 1.5 ml of 80% methanol was added to each 6 cm plate and cells were incubated at −80 °C for 1 h. Plates were scraped for cell harvesting and centrifuged at 4 °C. The supernatant was speed vac dried, and the metabolites were resuspended in 20 ml of LC/MS grade water and 5 ml were injected *via* targeted LC-MS/MS performed using a 6500 QTRAP triple quadrupole mass spectrometer (AB/SCIEX) coupled to a Prominence UFLC HPLC system (Shimadzu) with Amide HILIC chromatography (Waters). Data were acquired in selected reaction monitoring mode using positive/negative ion polarity switching for steady-state polar profiling of 294 molecules. Peak areas from the total ion current for each metabolite selected reaction monitoring transition were integrated using MultiQuant v3.0 software (AB/SCIEX).

## Data availability

All data supporting the findings of this study are available from the corresponding author upon request.

## Supporting Information

This article contains supporting information.

## Conflict of interest

The authors declare that they have no conflicts of interest with the contents of this article.

## Supporting information

Supplemental Figure S3

Supplemental Figure Captions
