# Supplementary material for: Prolonged deprivation of arginine or leucine induces PI3K/Akt-dependent reactivation of mTORC1
Source: J Biol Chem. 2022 May 13;298(6):102030. doi: 10.1016/j.jbc.2022.102030 (PMC9194872; doi:10.1016/j.jbc.2022.102030)
Supplement: File S1 [file mmc1.pdf]

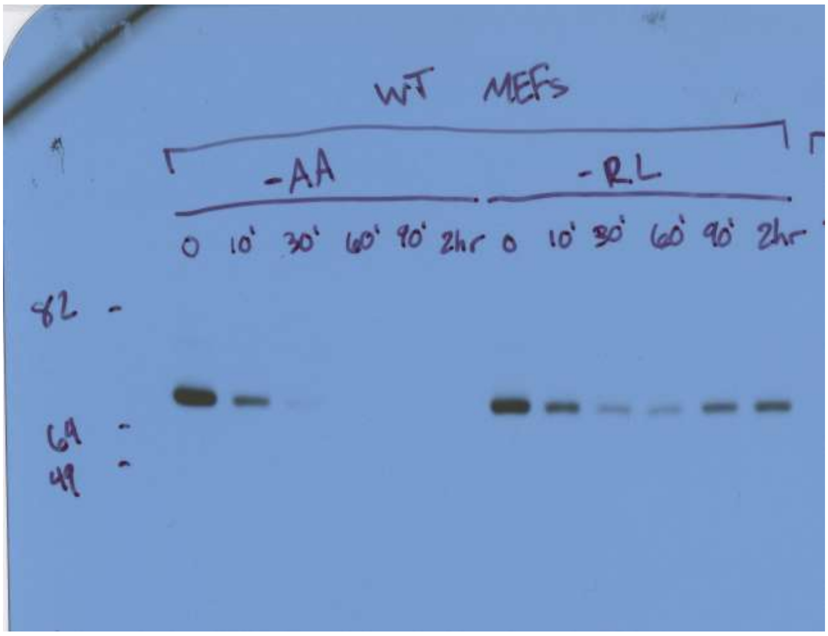

p-S6K1 (T389)

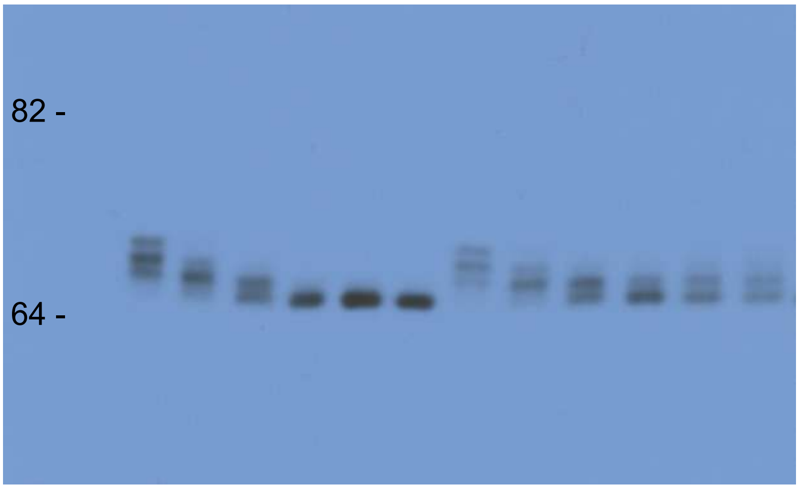

S6K1

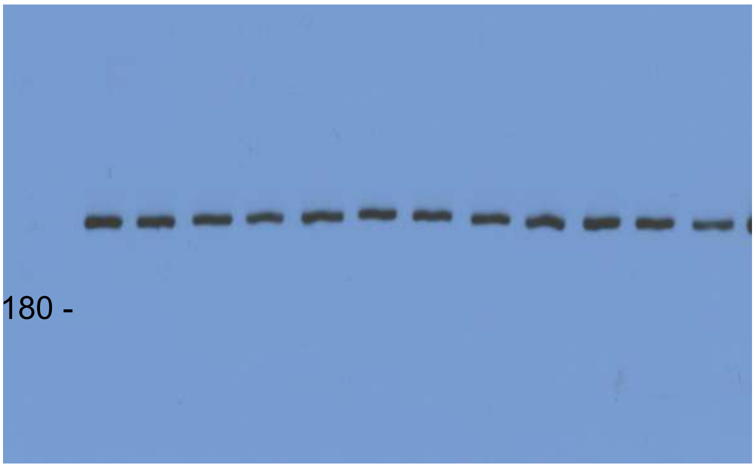

mTOR

Fig 1A Raw data

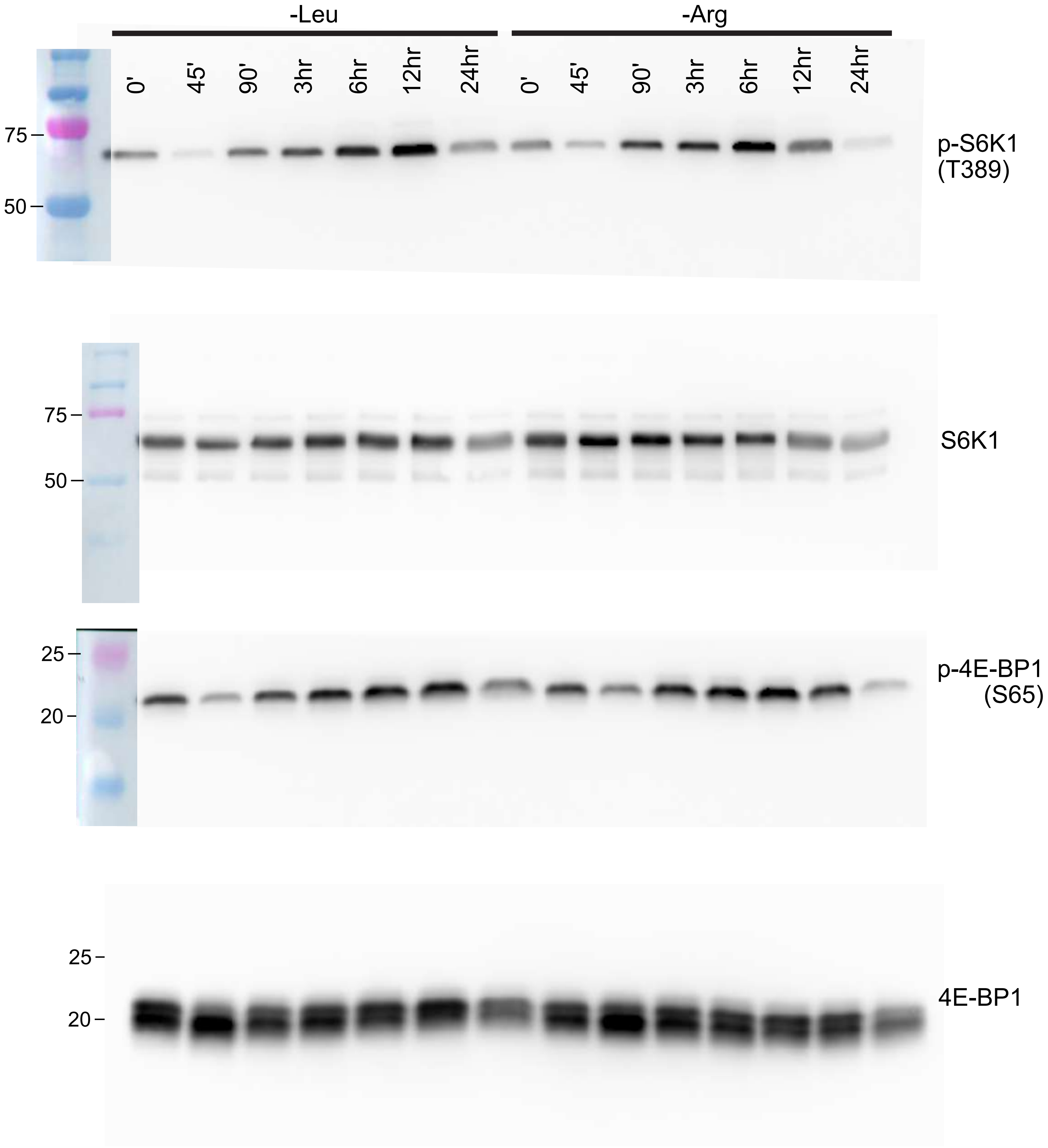

Fig 1B Raw data

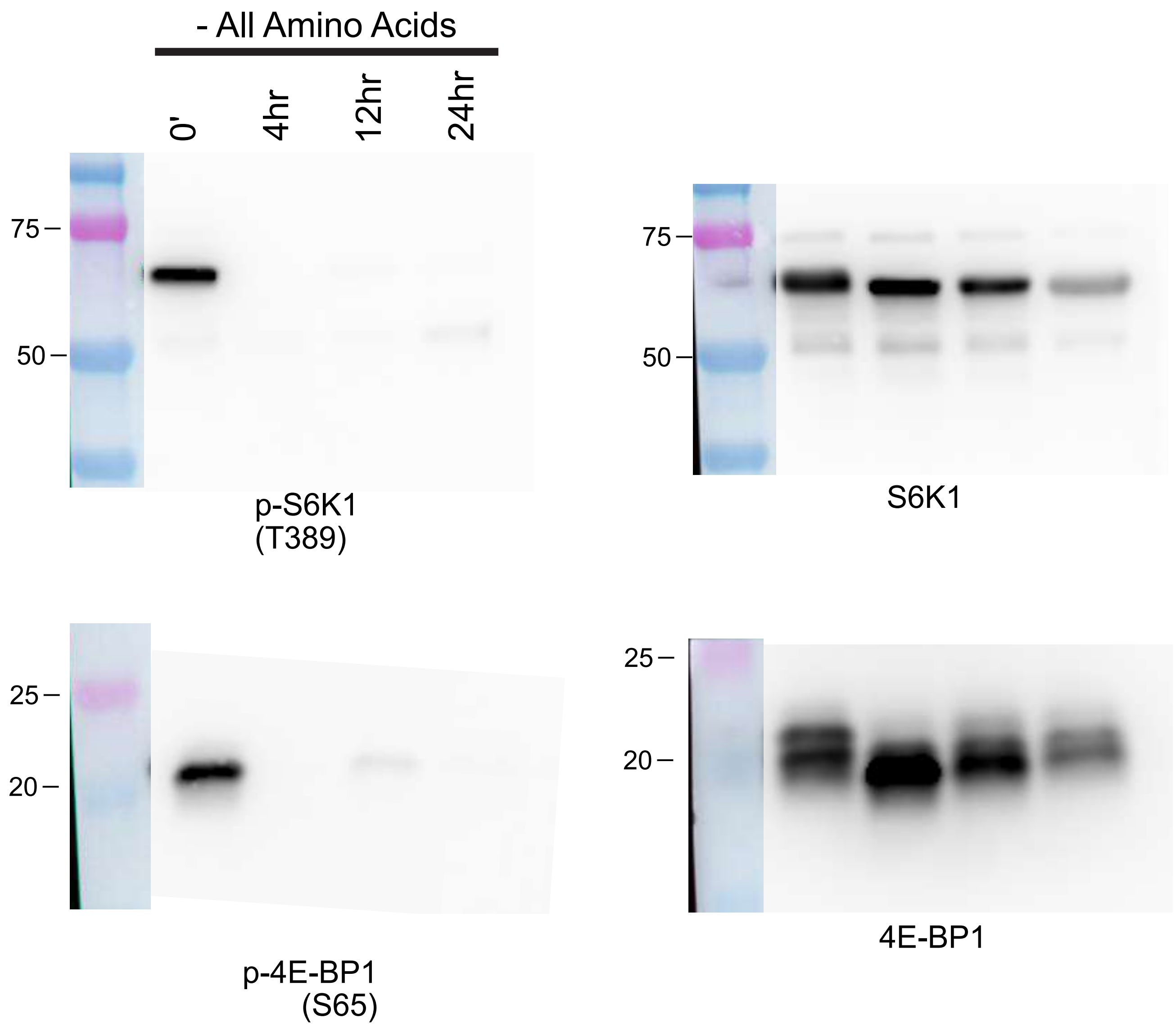

Fig 1E Raw data

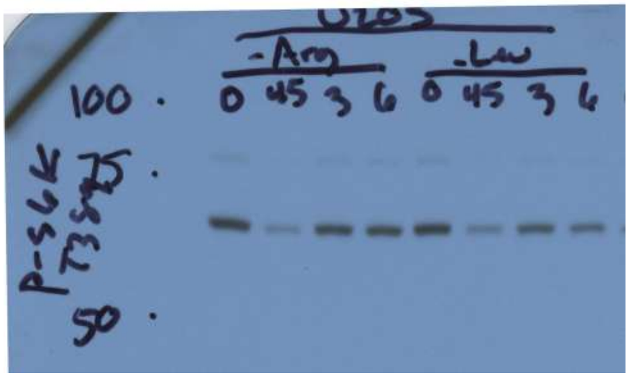

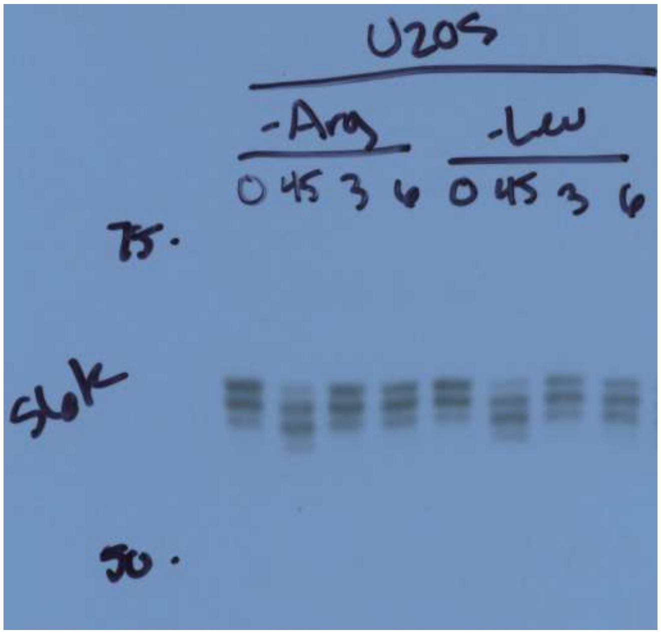

Fig 2A Raw data

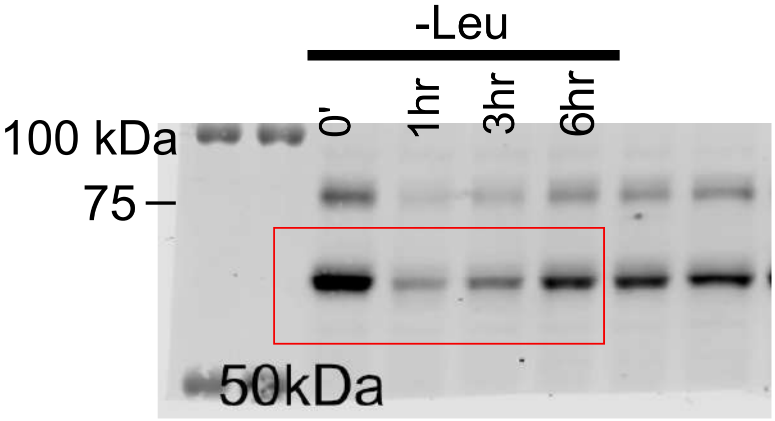

p-S6K T389

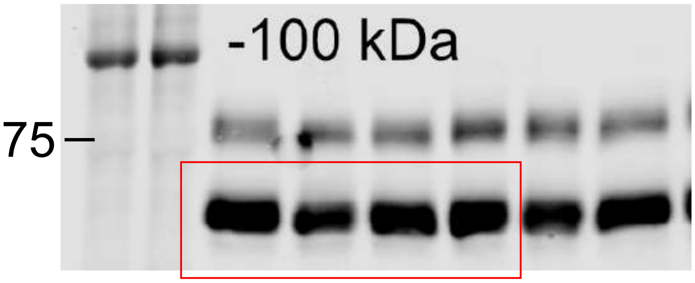

tot S6K

Fig 2B Raw data

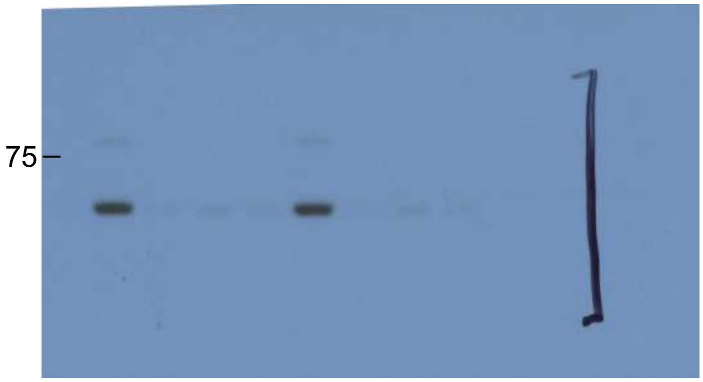

p-S6K1
(T389)

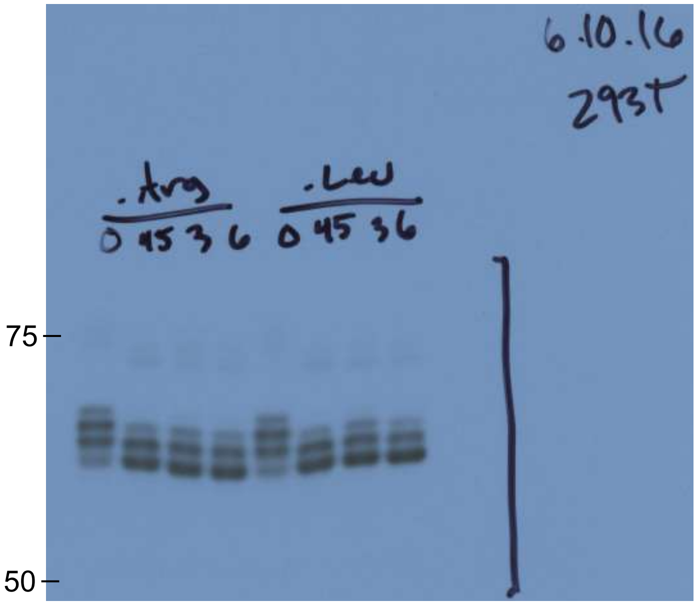

S6K1

Fig 2C Raw data

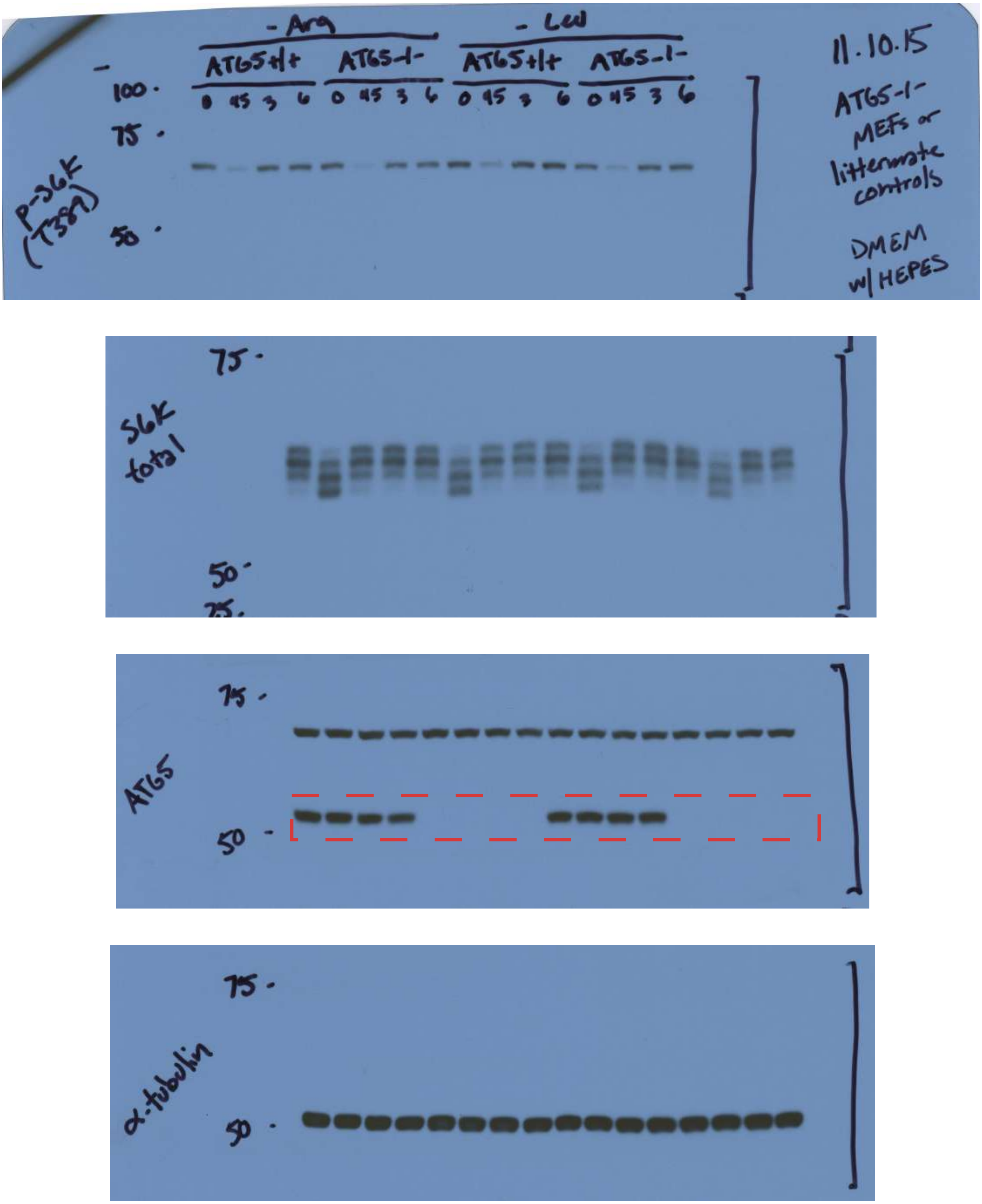

Fig 3A Raw data

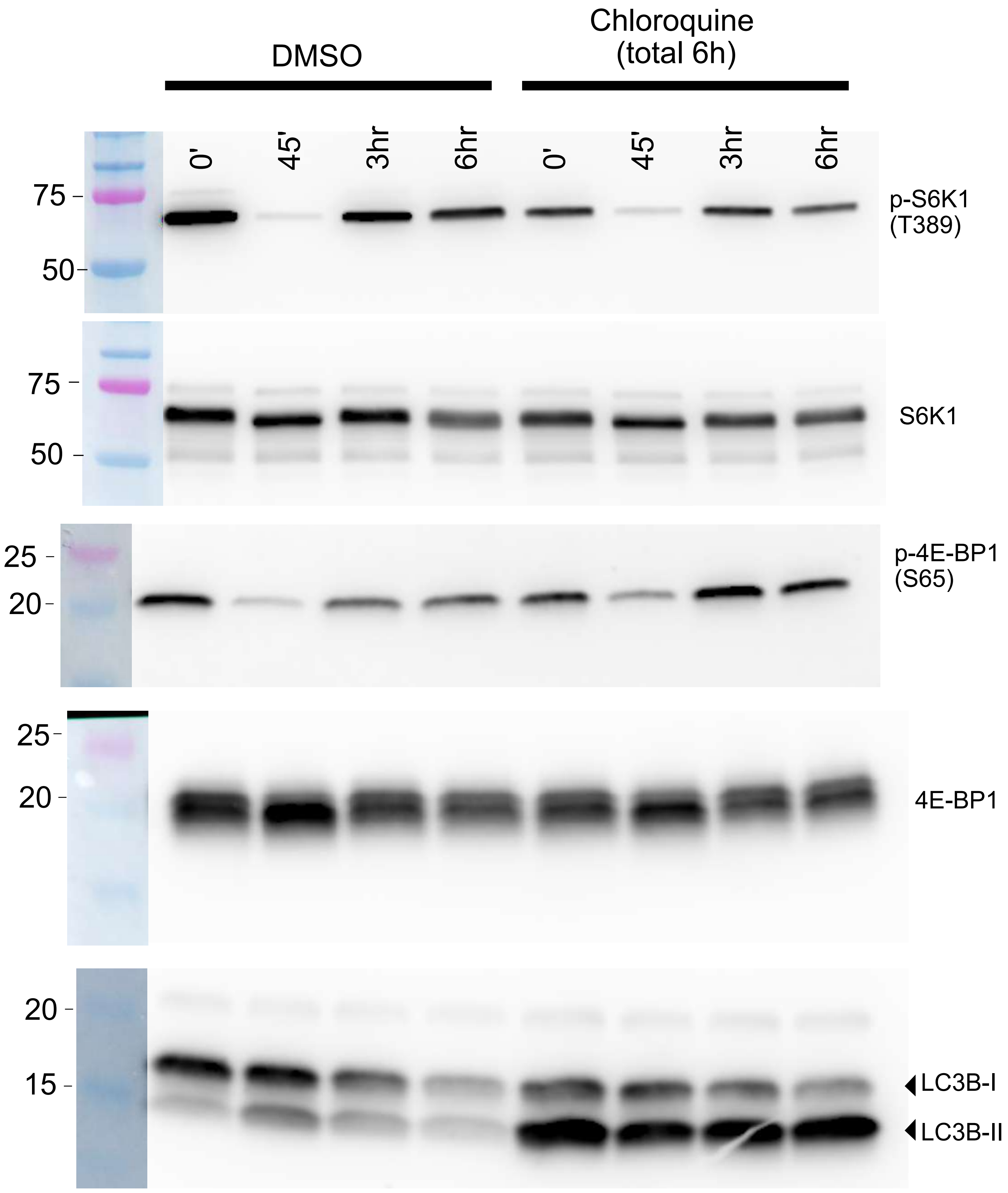

Fig 3B Raw data

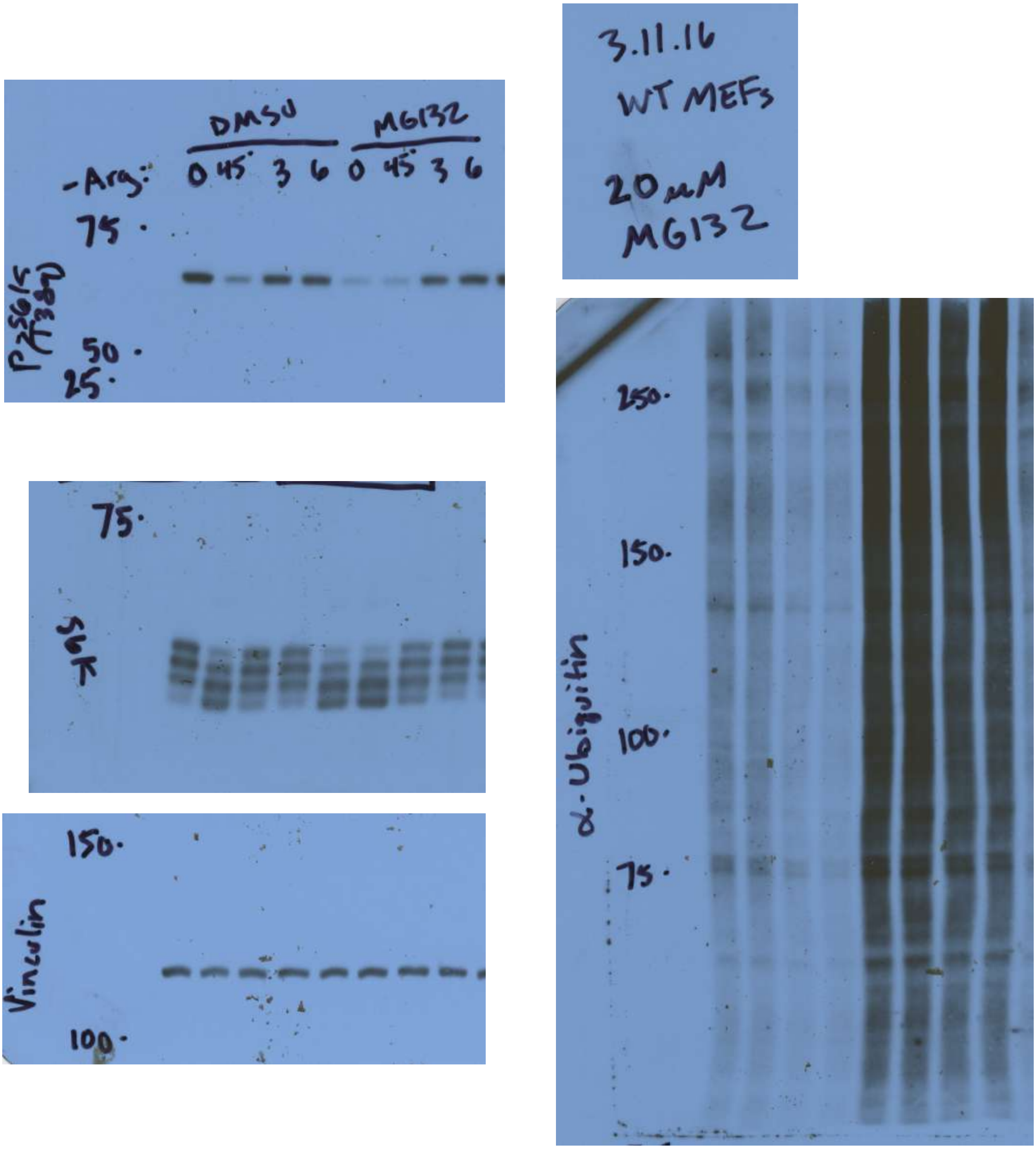

Fig 3C Raw data

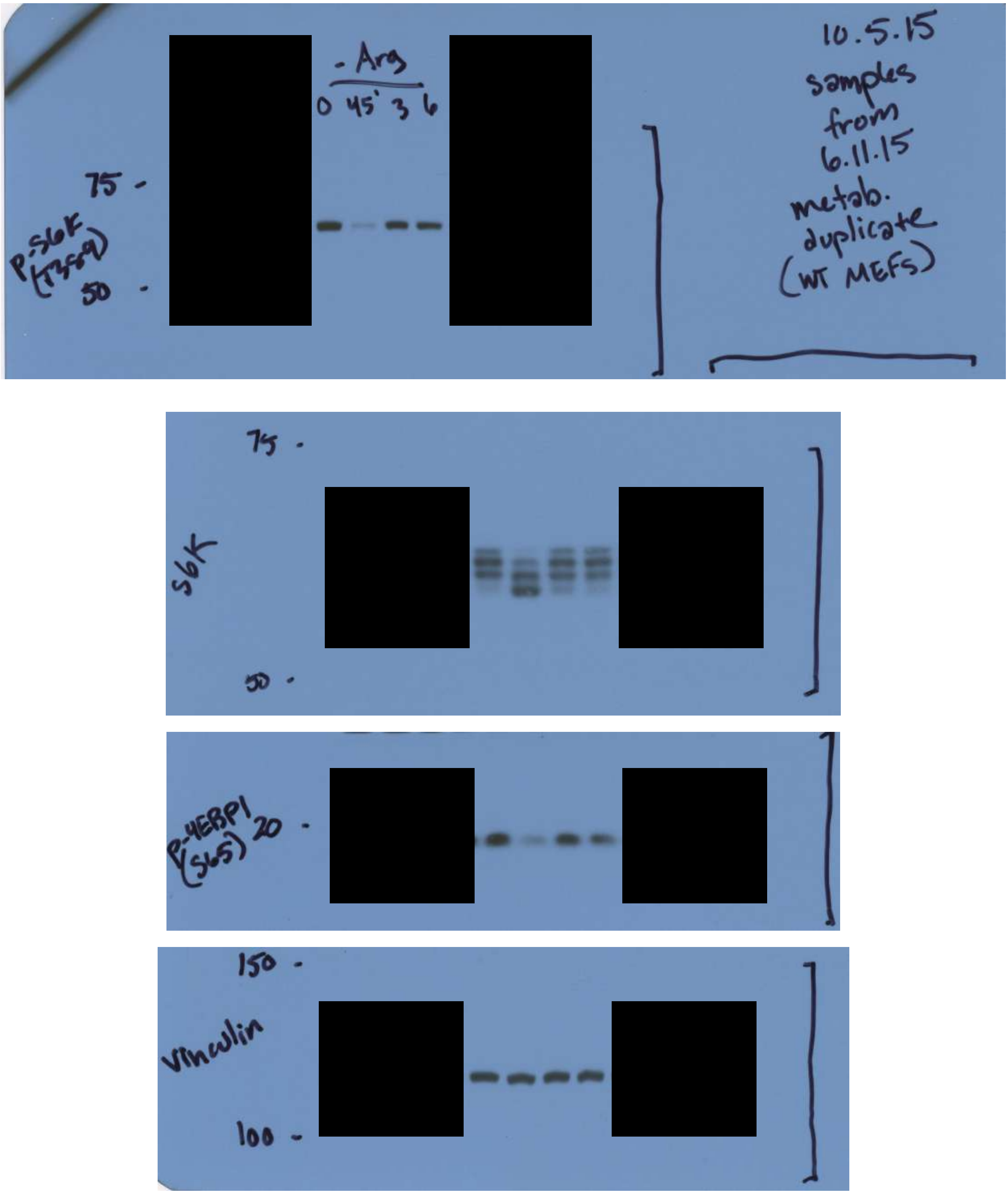

Fig 3D Raw data

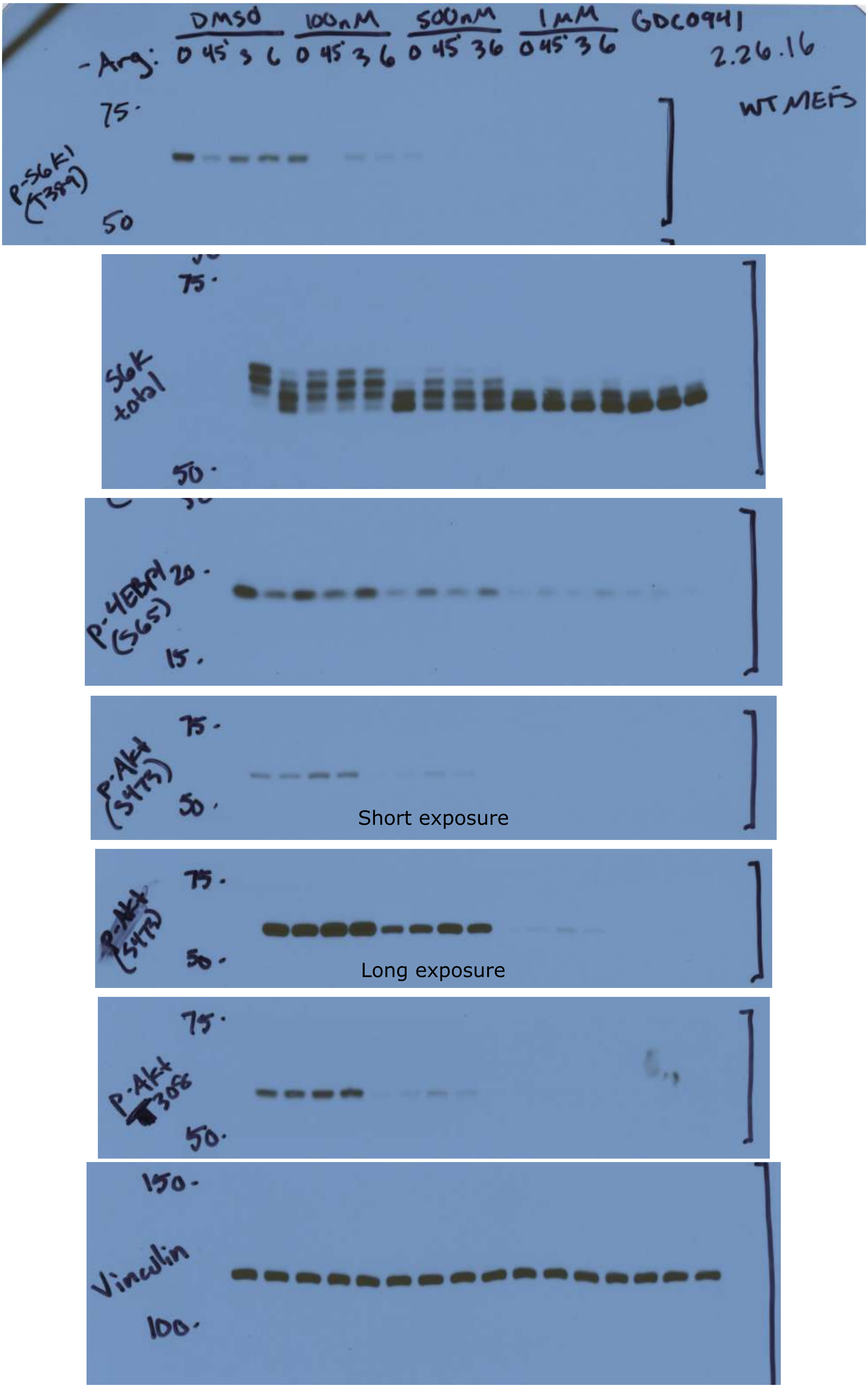

Fig 4A Raw data

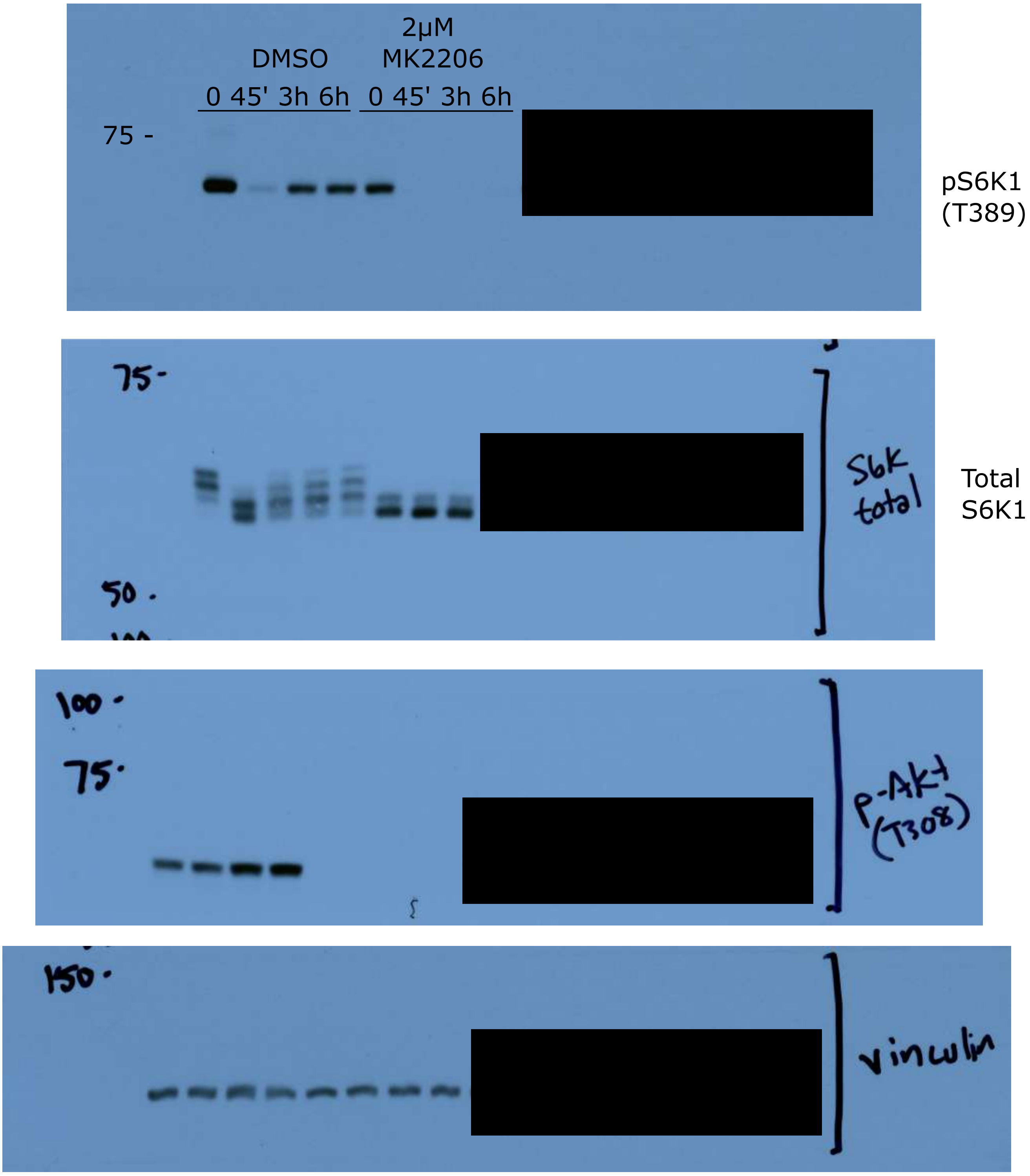

Fig 4B Raw data

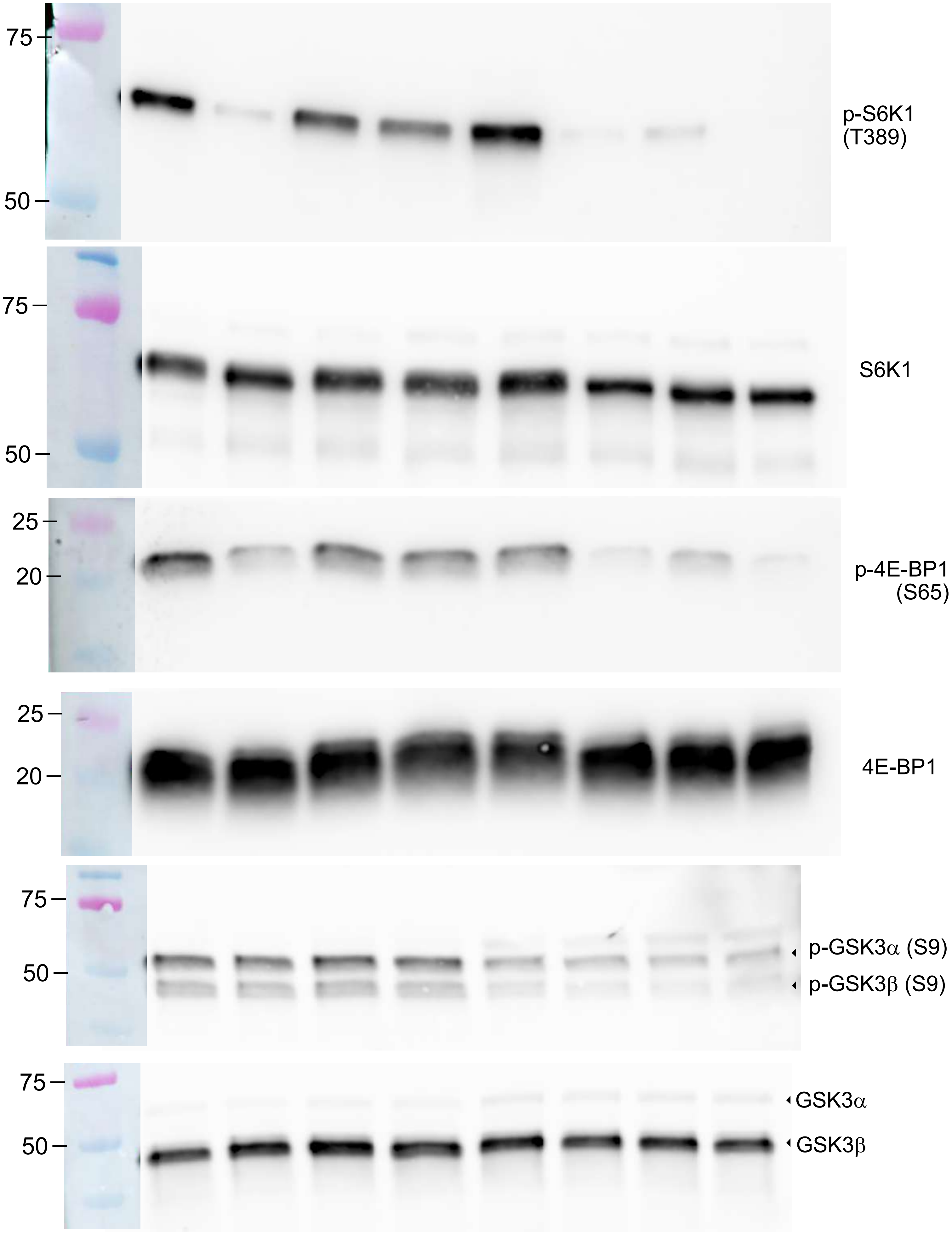

Fig 4C Raw data

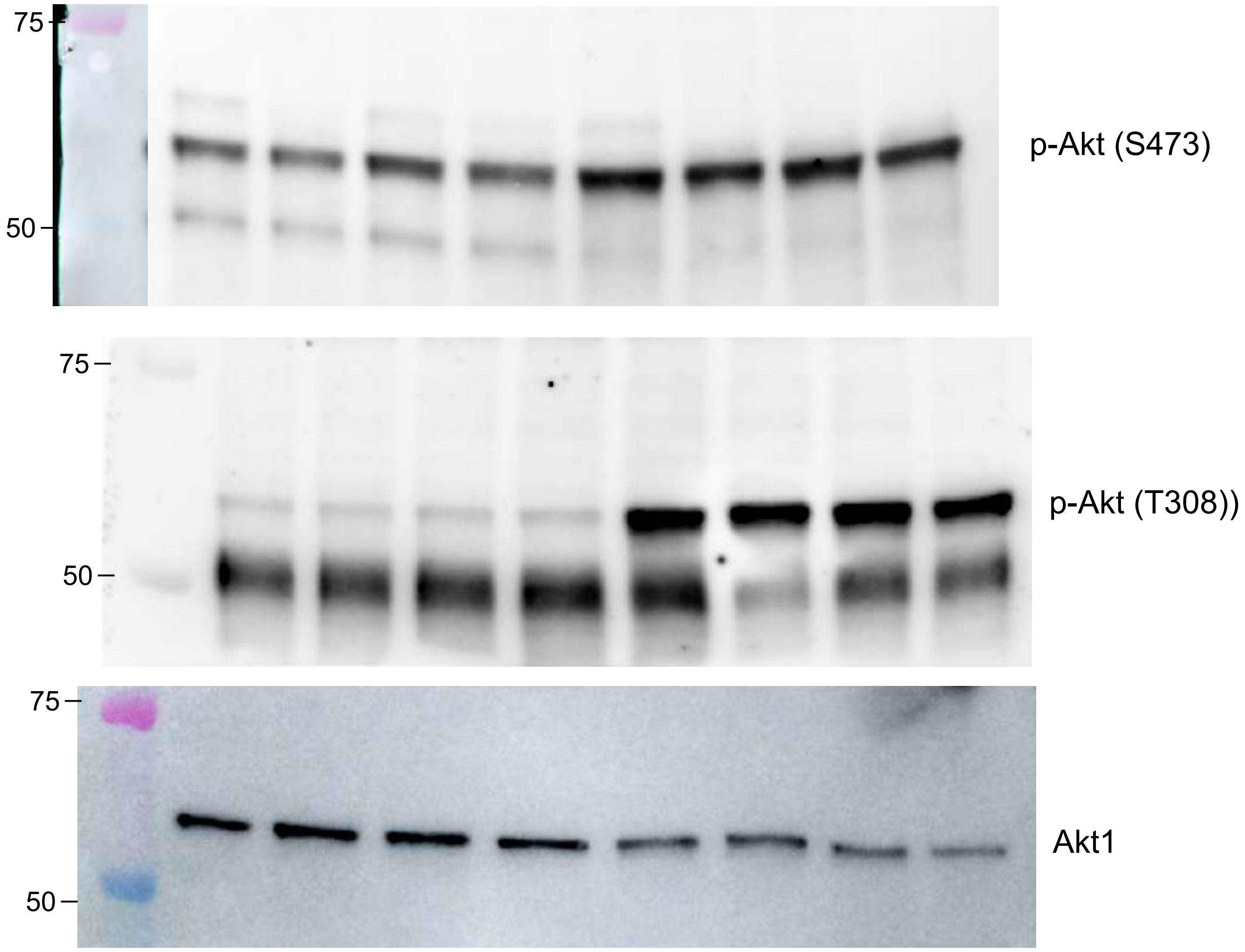

Fig 4C Raw data(Cont)

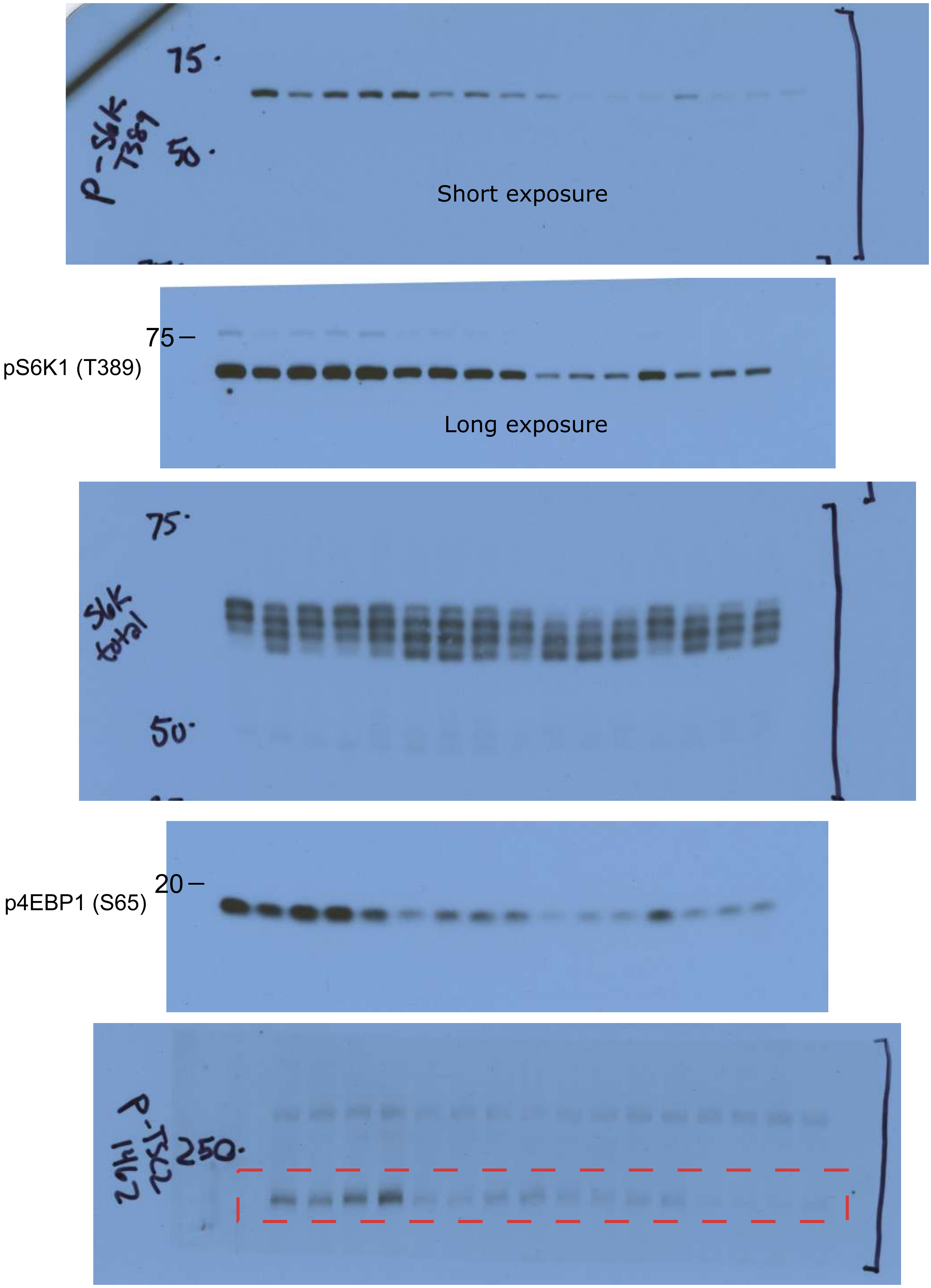

Fig 4D Raw data

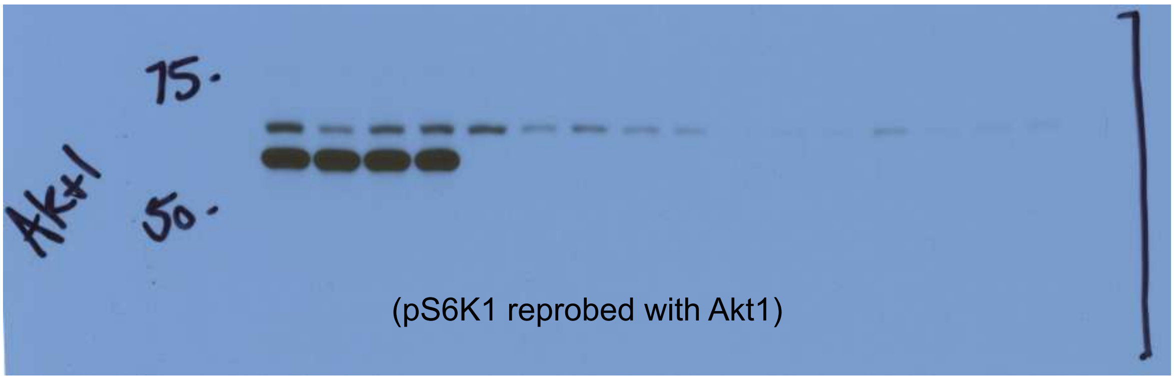

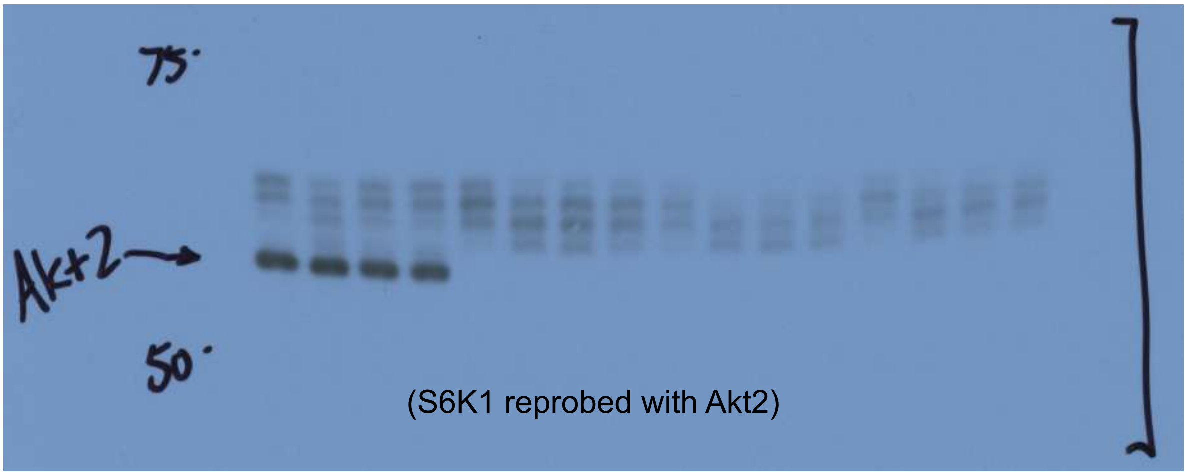

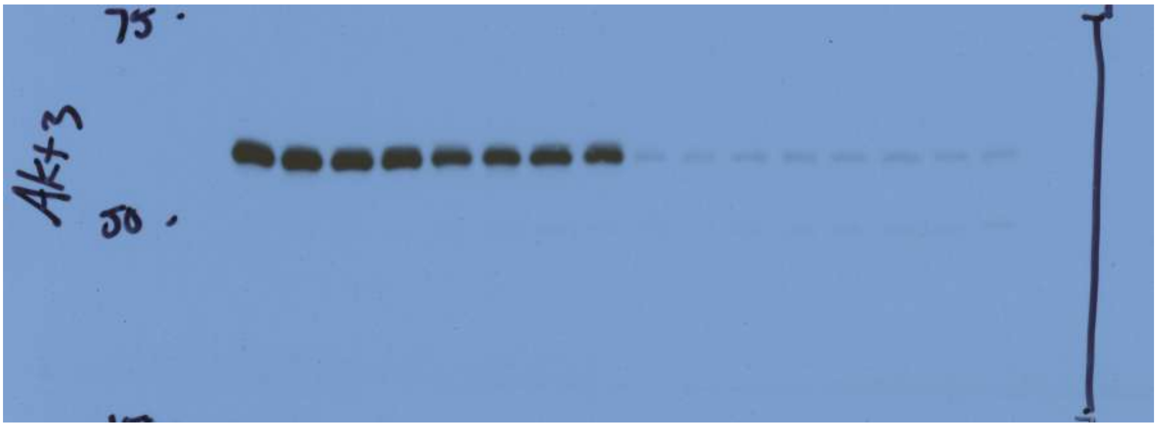

Vinculin

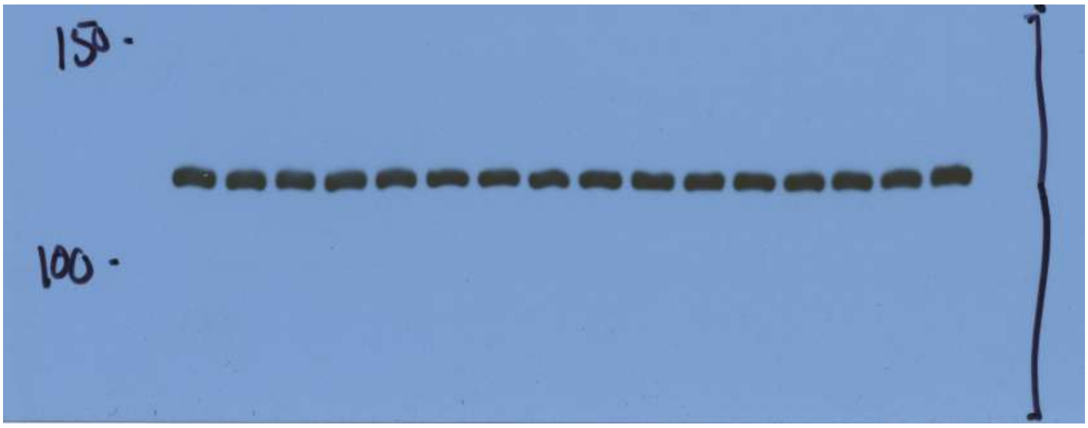

Fig 4D Raw data cont

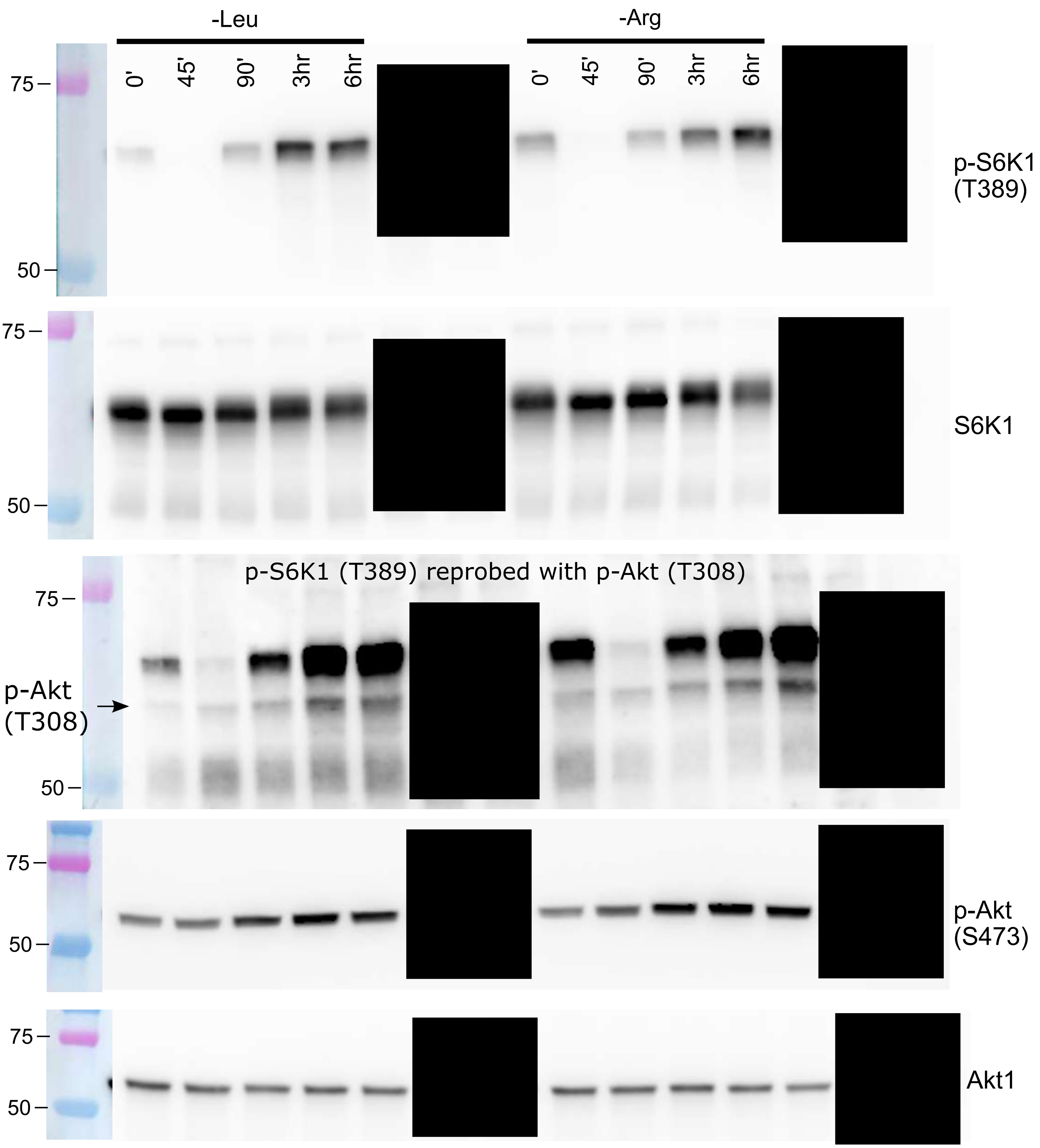

Fig 5A Raw data

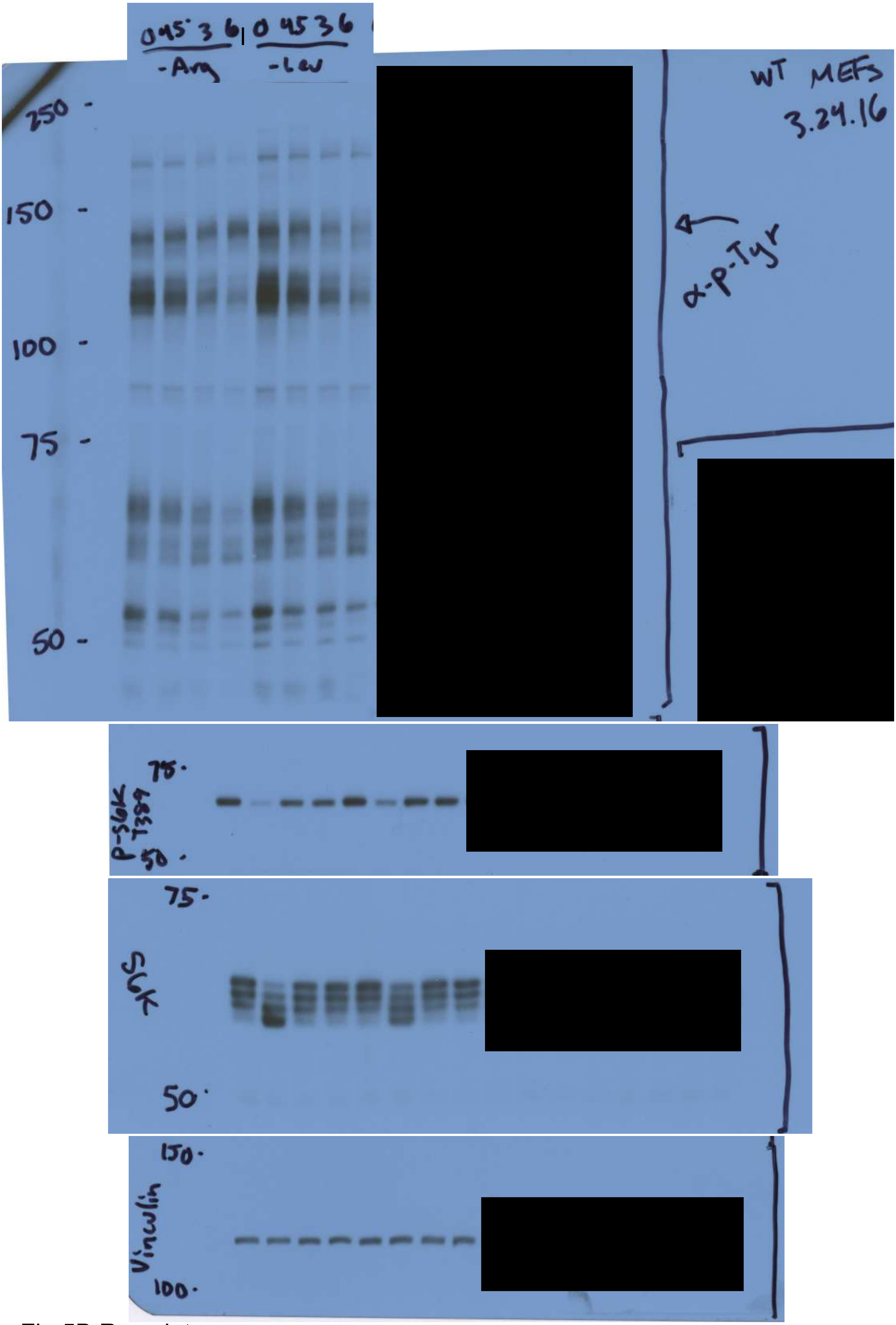

Fig 5B Raw data

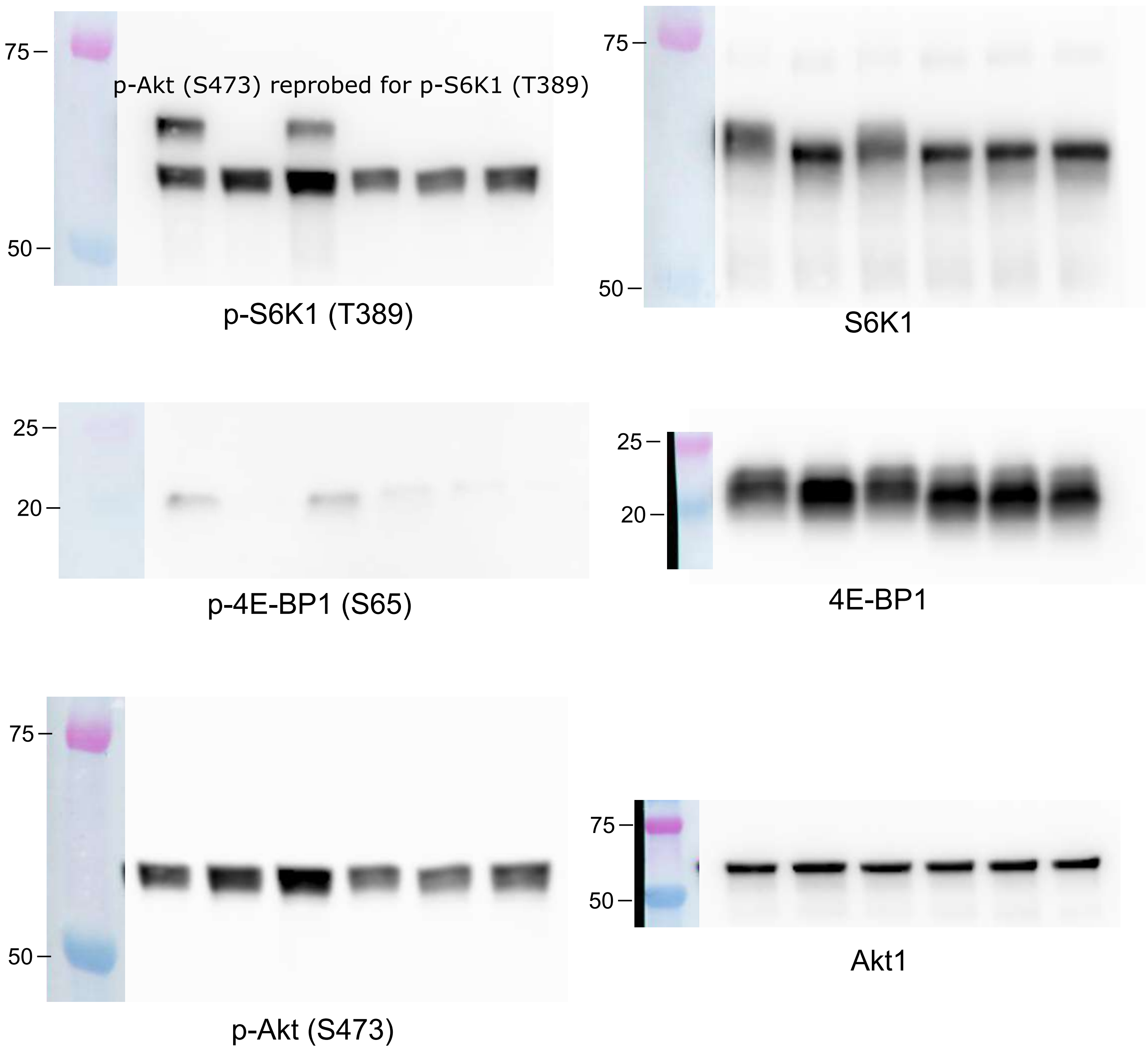

Fig 5C Raw data

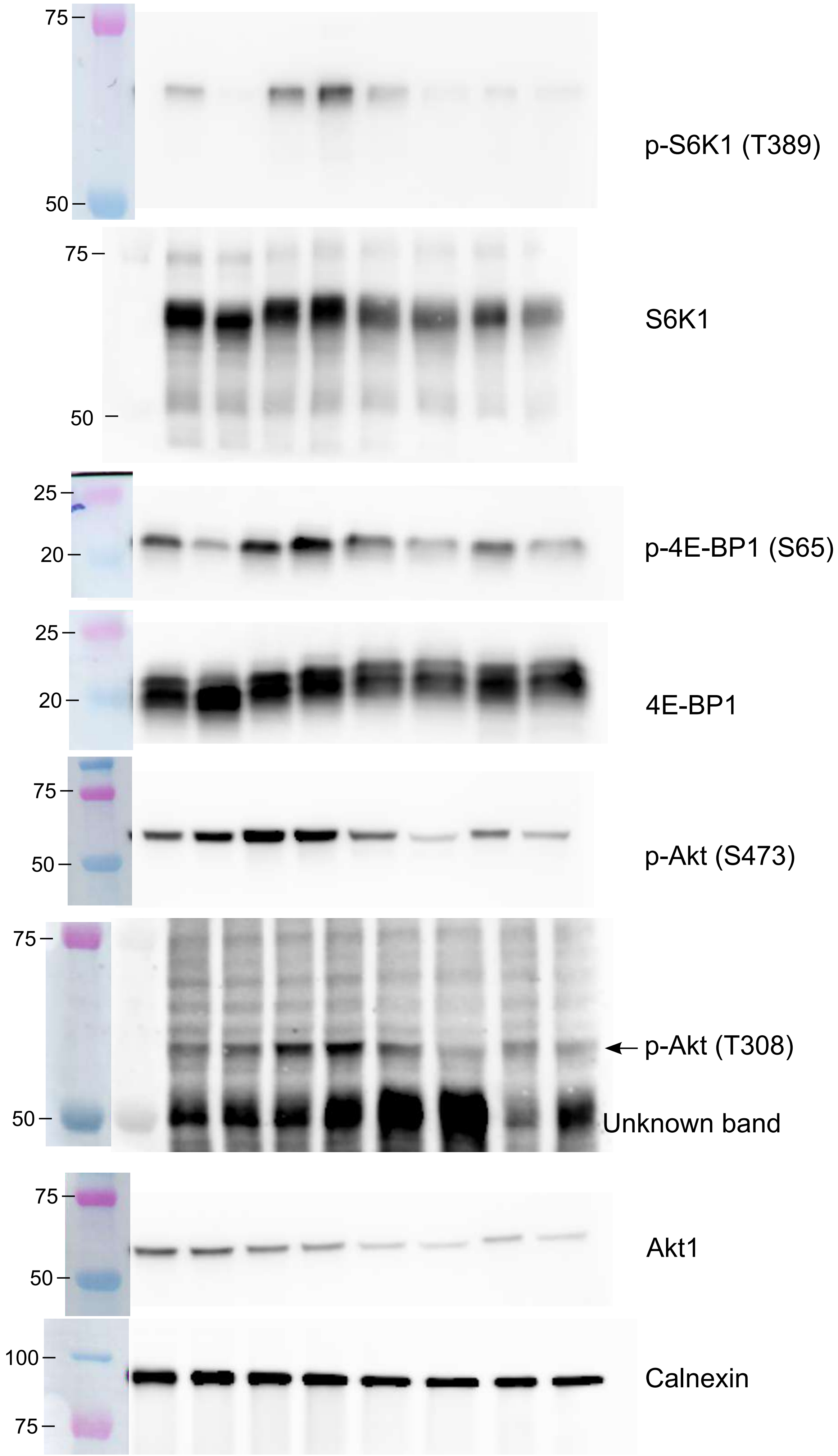

Fig 6A Raw data

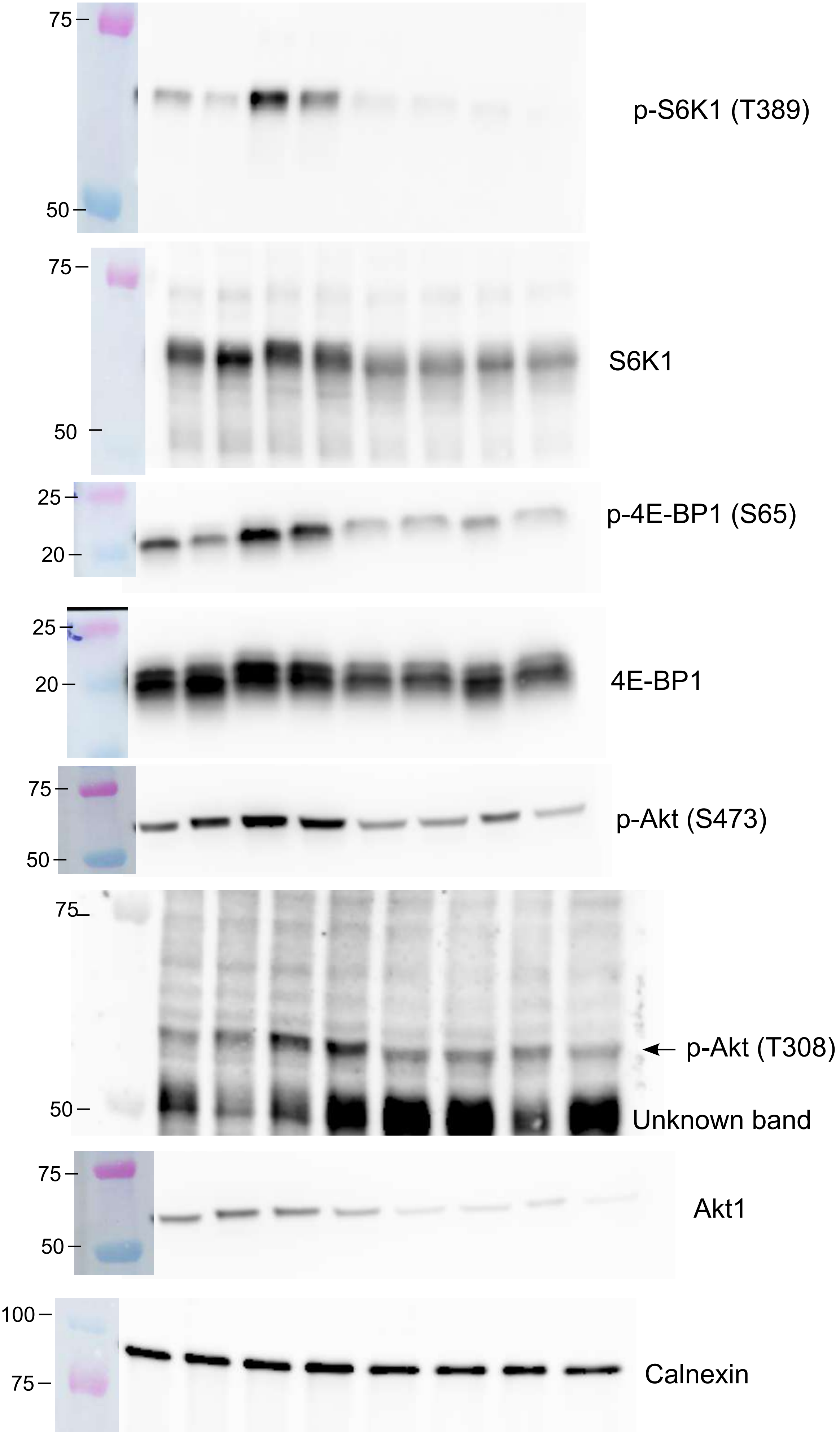

Fig 6B Raw data

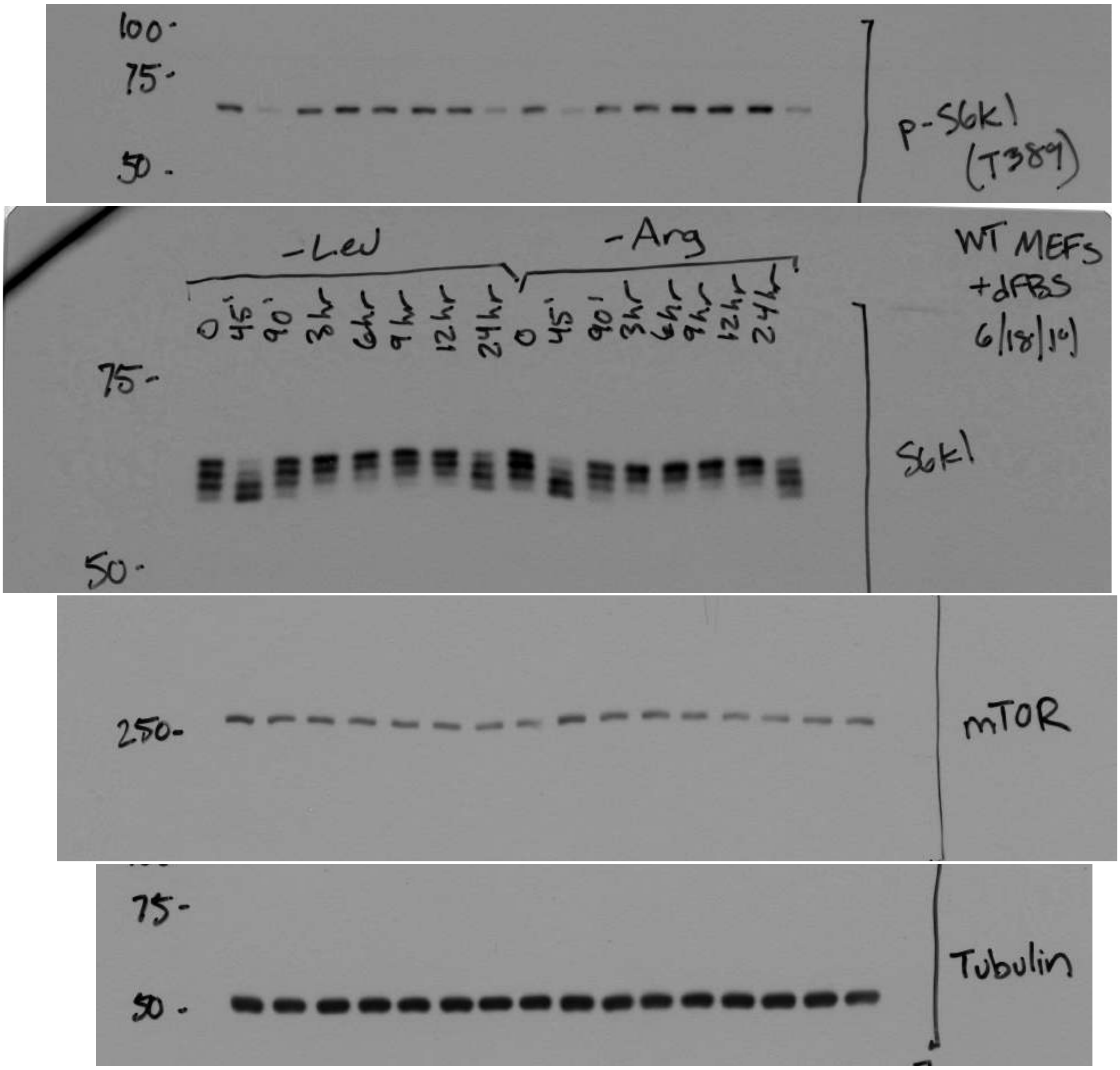

Fig S1A Raw data

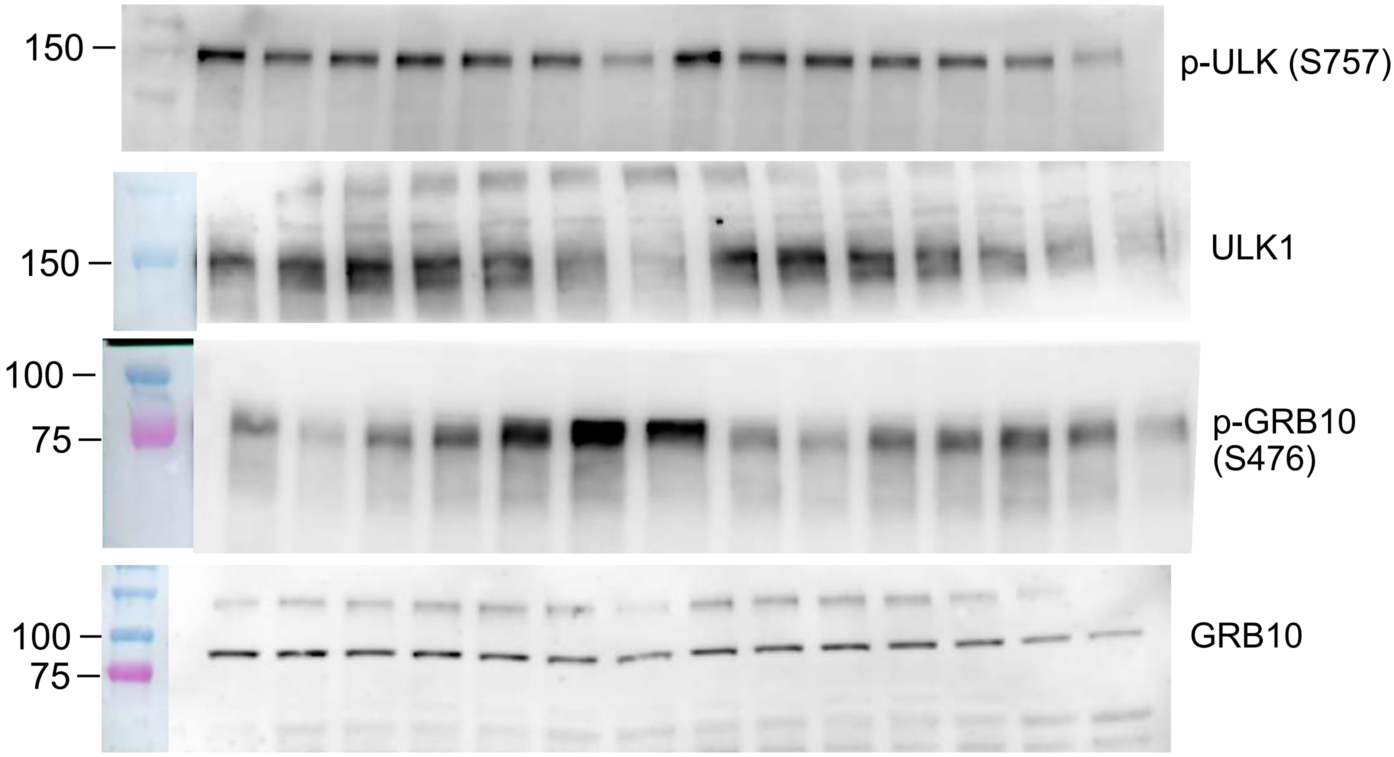

Fig S1B Raw data

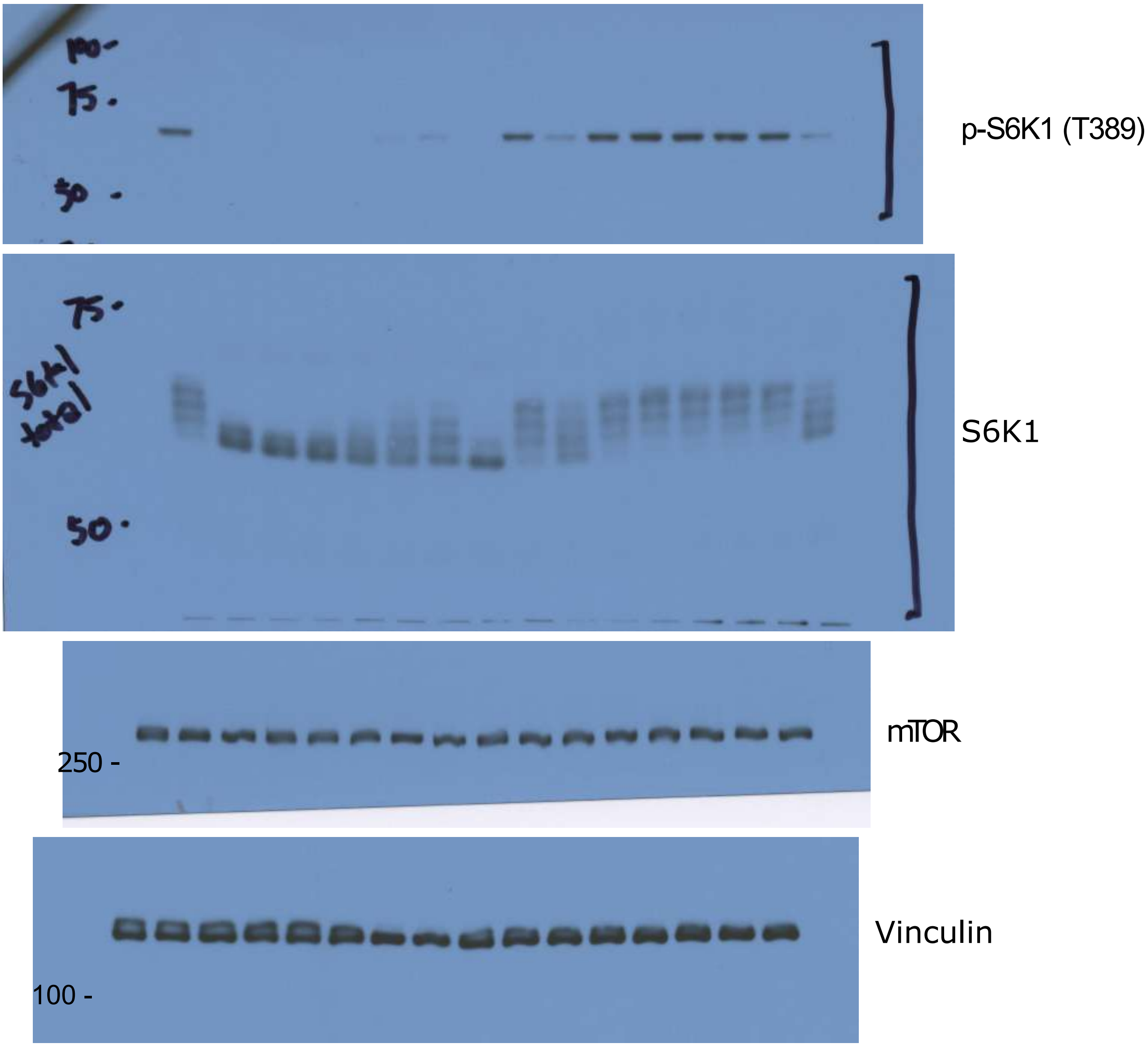

Fig S1C Raw data

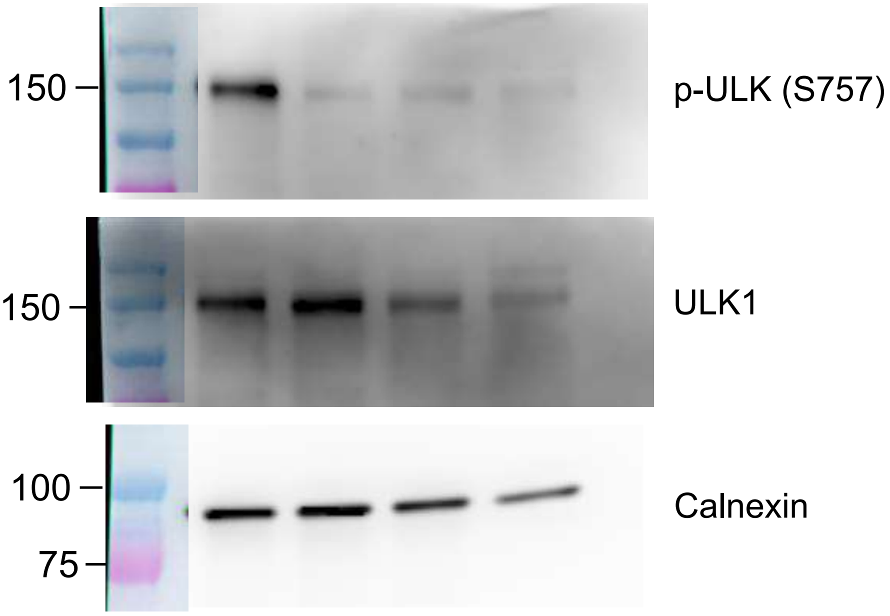

Fig S1D Raw data

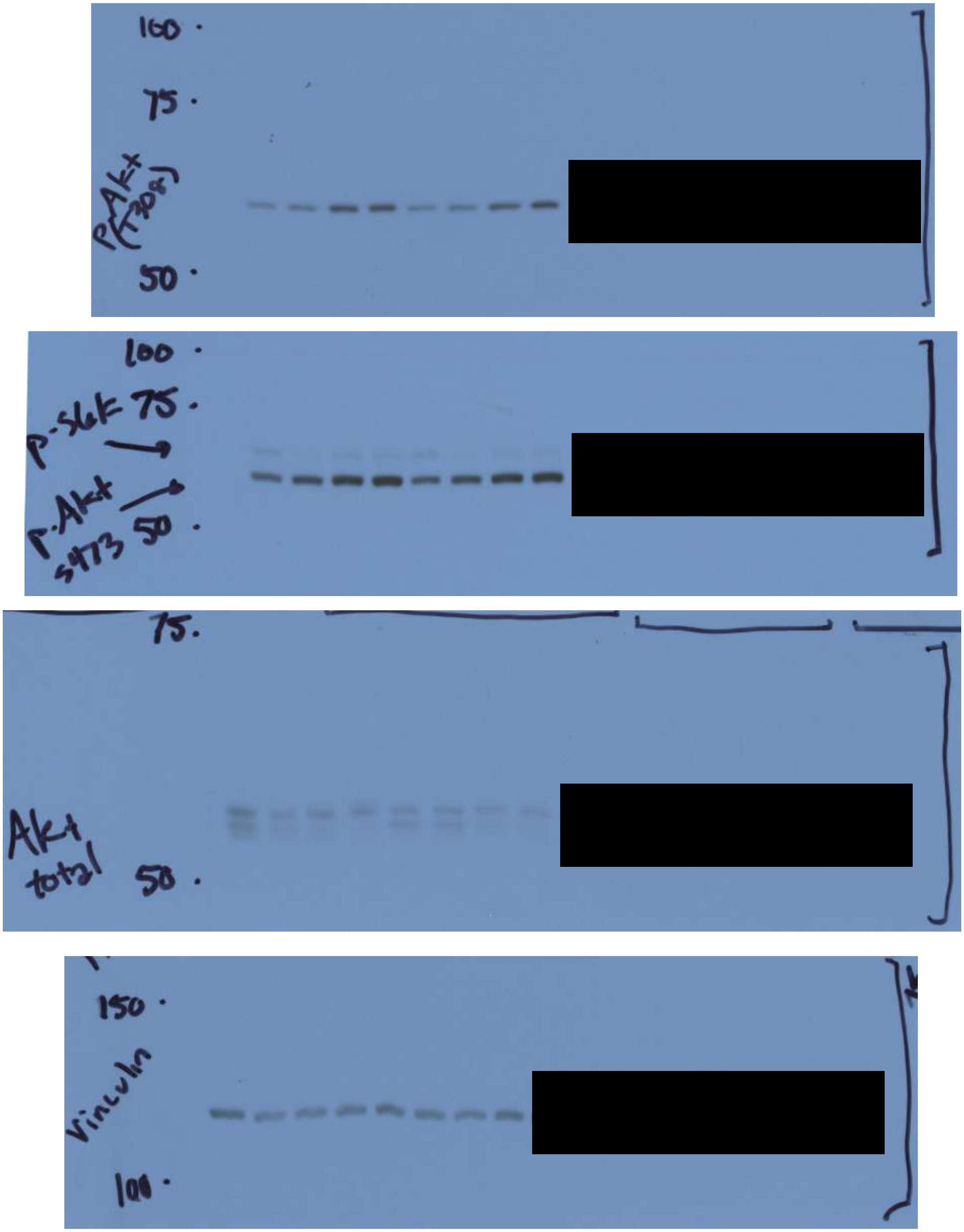

Fig S2A Raw data

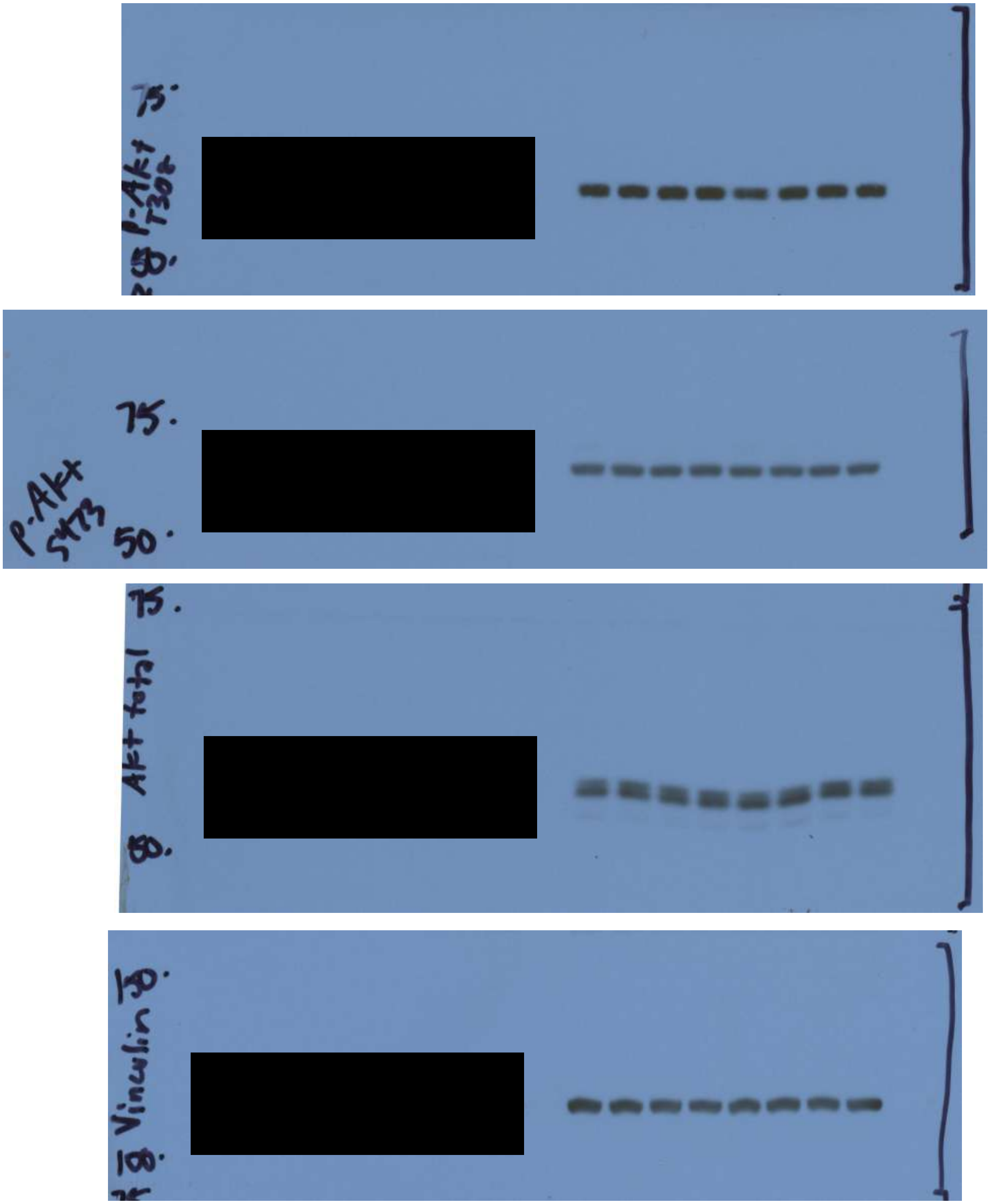

Fig S2B Raw data

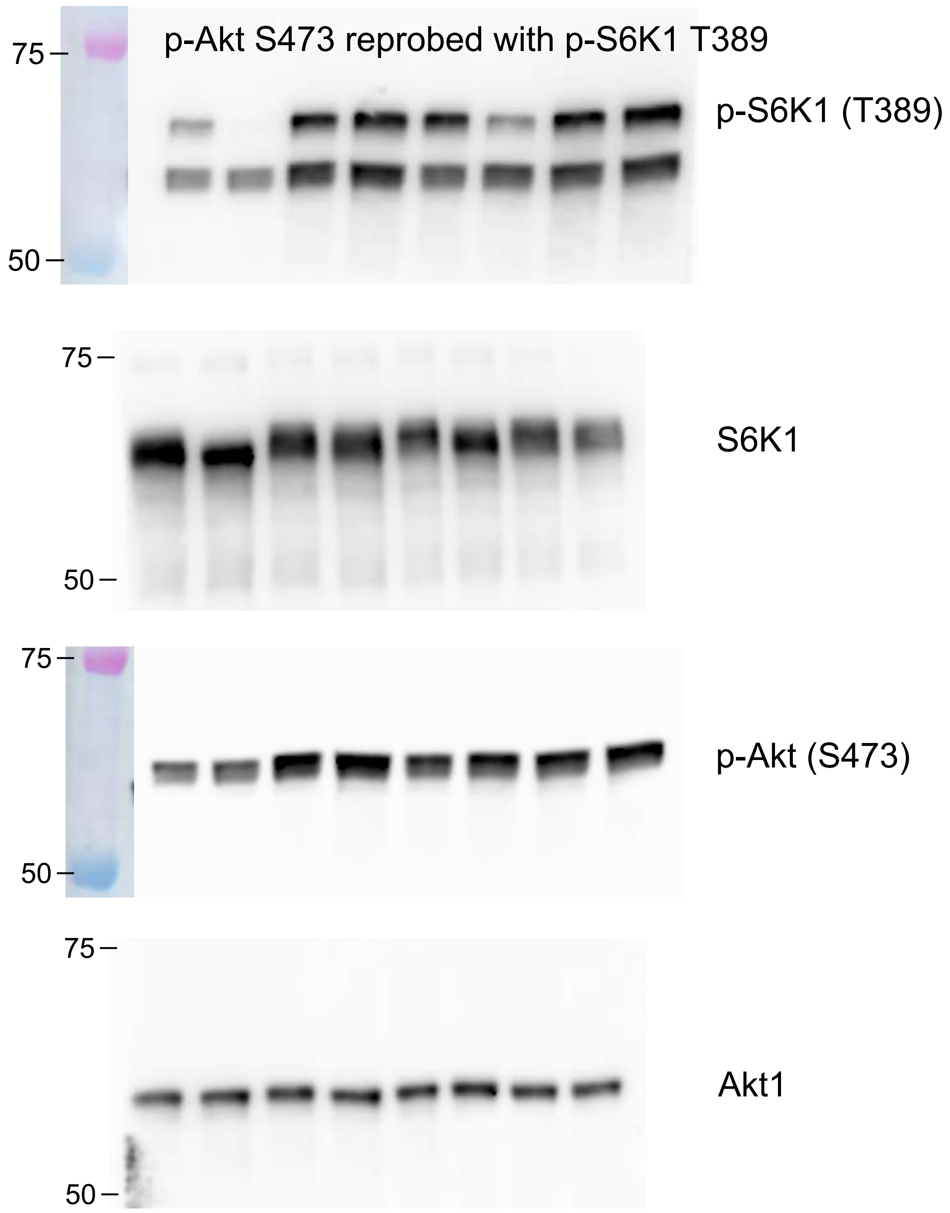

Fig S3B Raw data

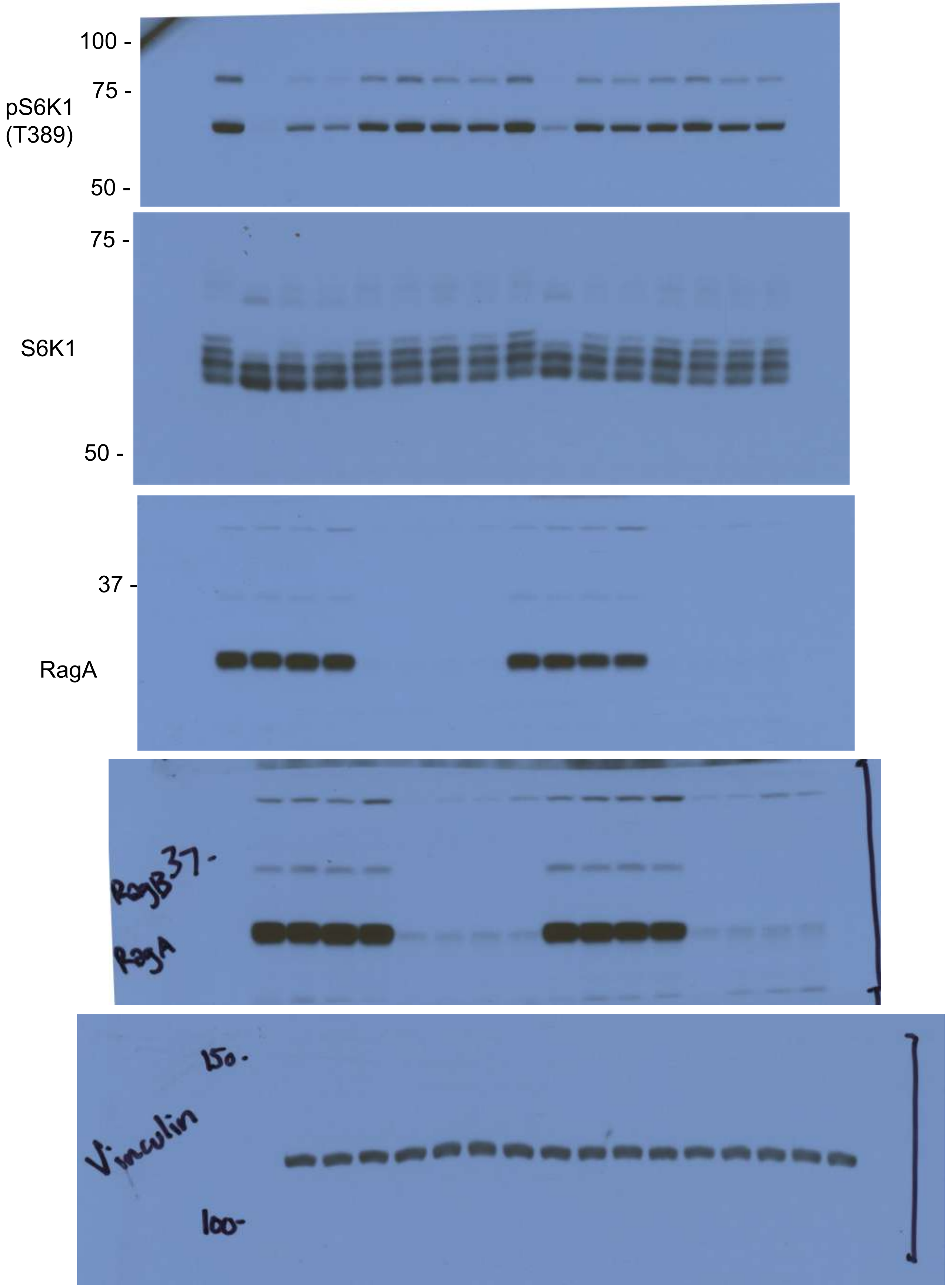

Fig S3C Raw data

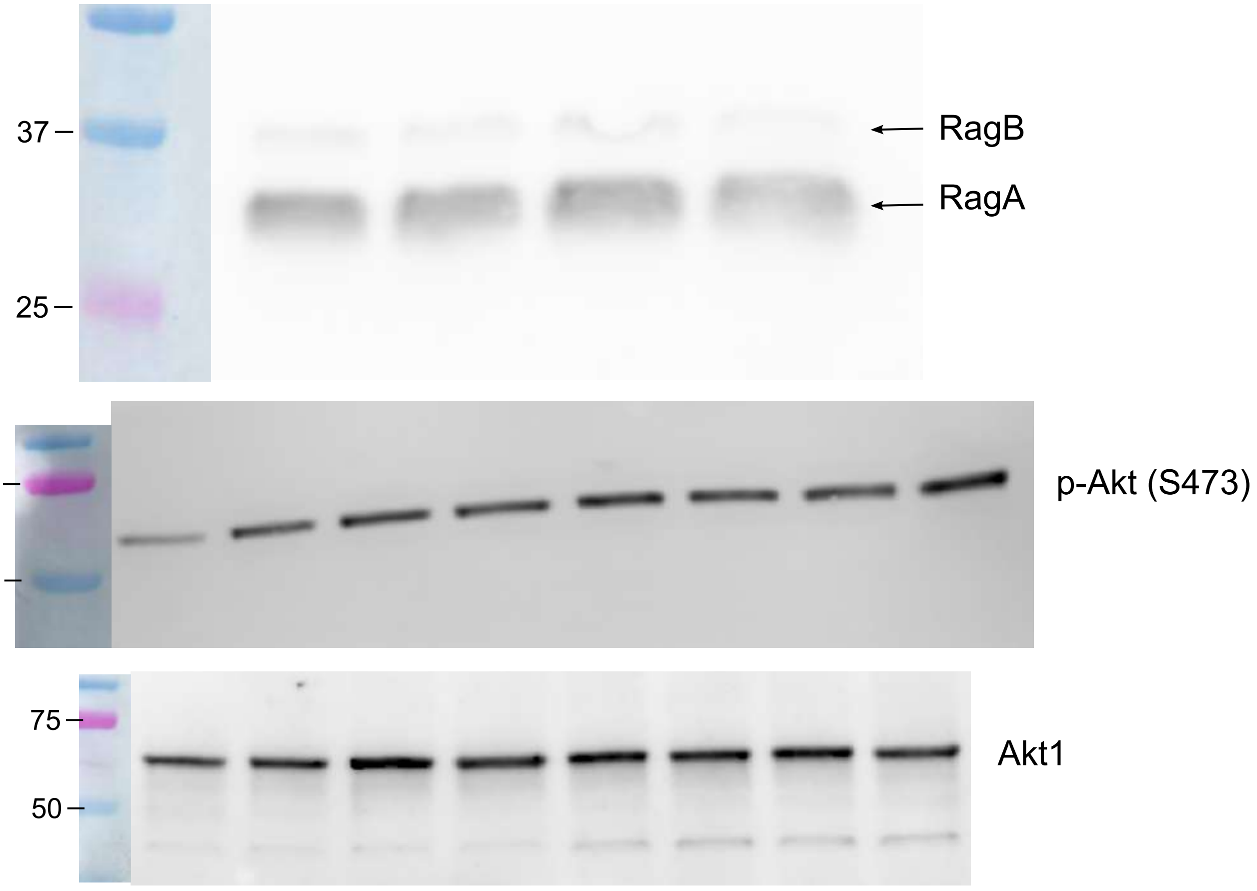

Fig S3D Raw data
