# Supplementary figures and images for: Prolonged deprivation of arginine or leucine induces PI3K/Akt-dependent reactivation of mTORC1
Source: J Biol Chem. 2022 May 13;298(6):102030. doi: 10.1016/j.jbc.2022.102030 (PMC9194872; doi:10.1016/j.jbc.2022.102030)

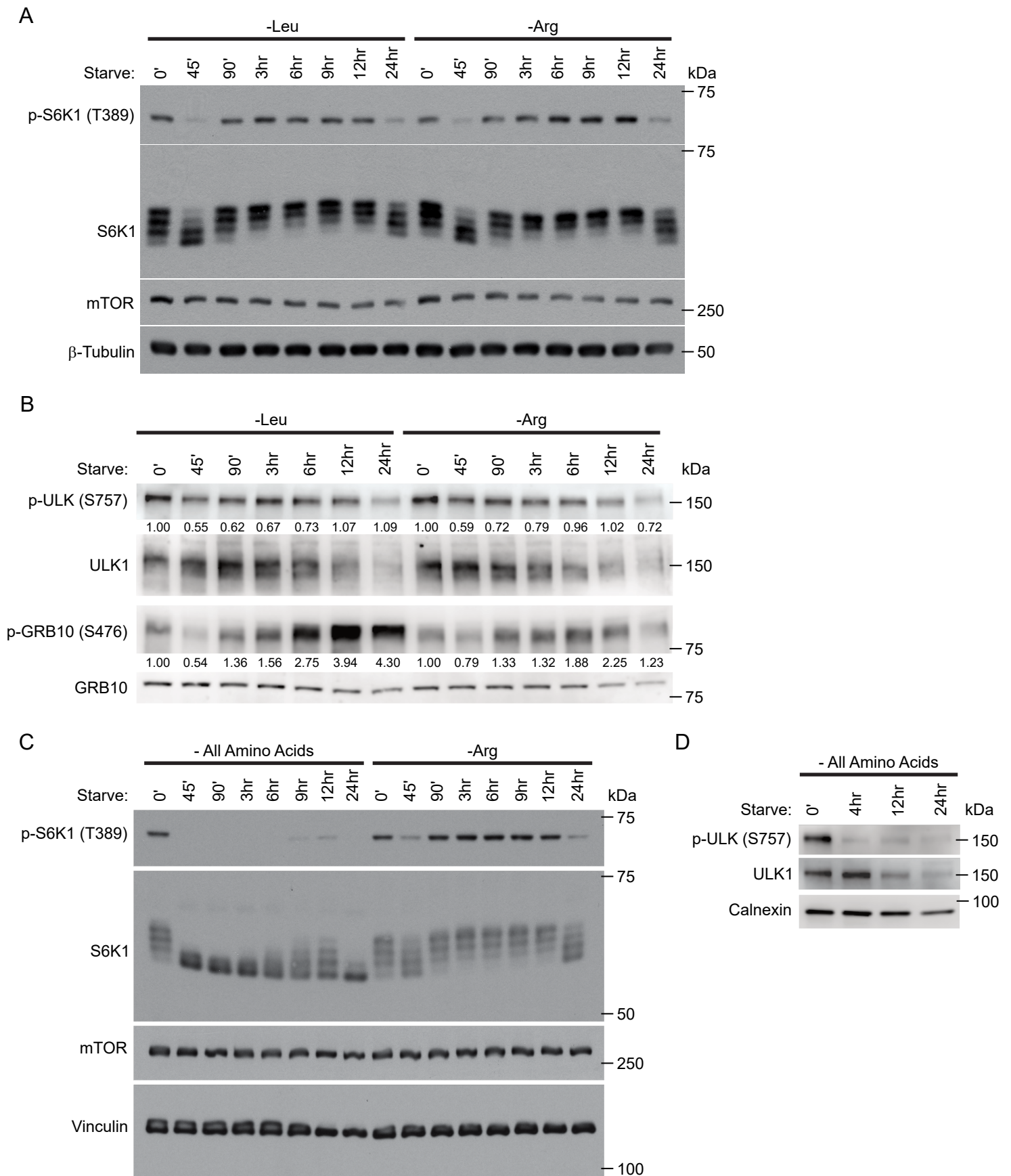

Figure S1

Supplement: Supplemental Figure S1 [file mmc2.pdf]

A

U2OS

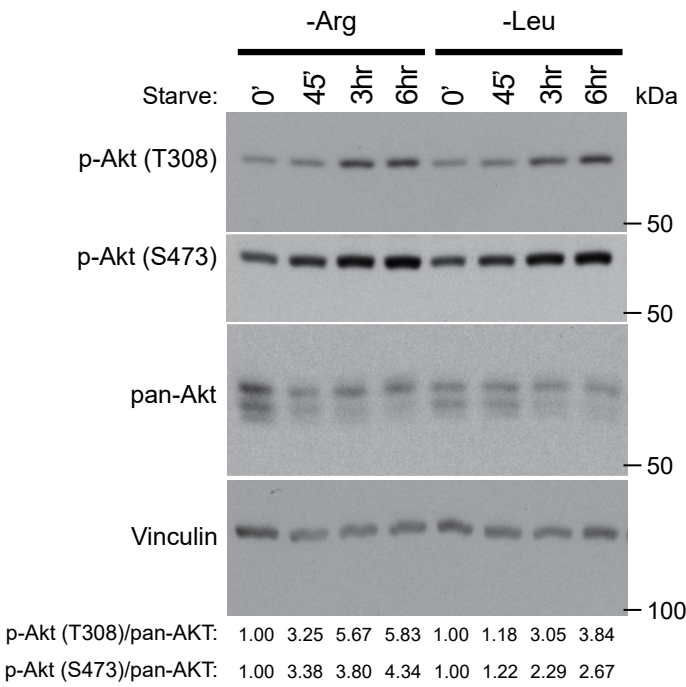

B

293T

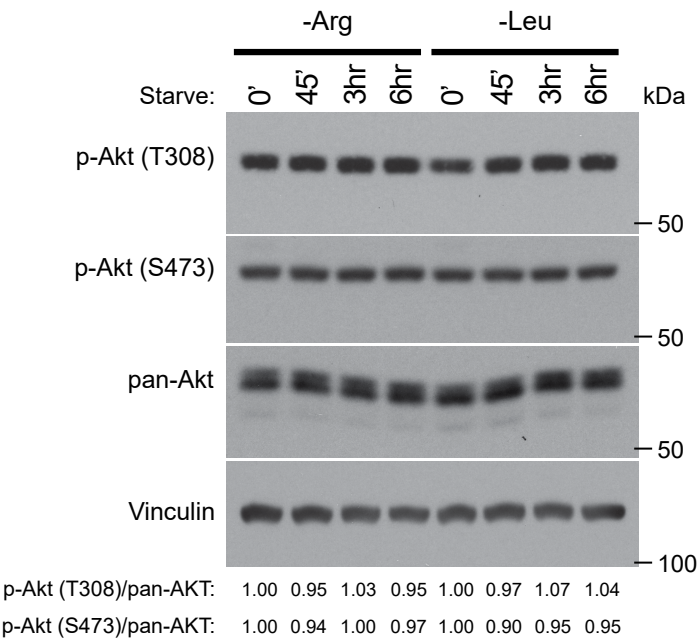

Figure S2

Supplement: Supplemental Figure S2 [file mmc3.pdf]

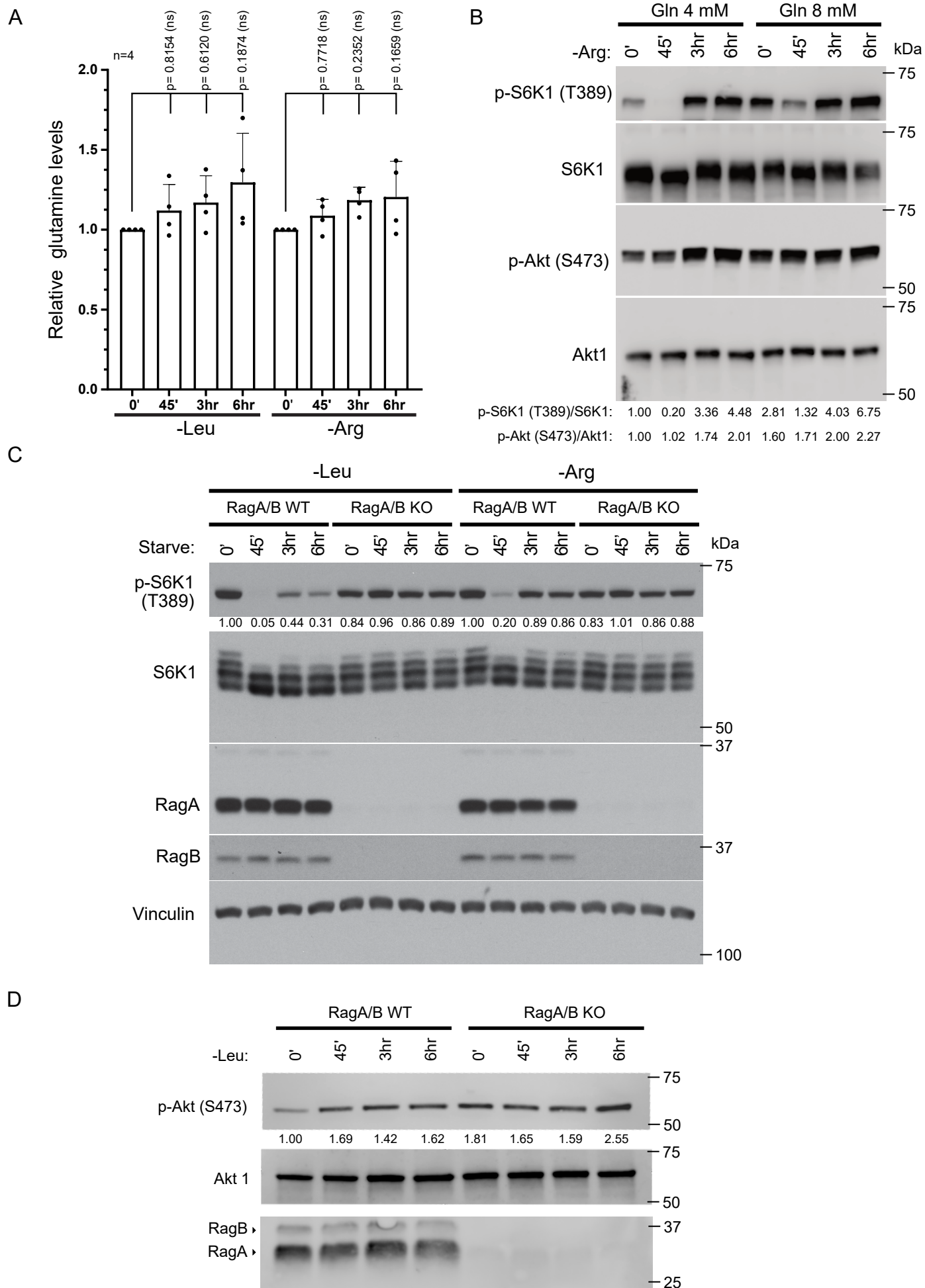

Figure S3

Supplement: Supplemental Figure S3 [file mmc4.pdf]
