# Supplementary material for: Prolonged deprivation of arginine or leucine induces PI3K/Akt-dependent reactivation of mTORC1
Source: J Biol Chem. 2022 May 13;298(6):102030. doi: 10.1016/j.jbc.2022.102030 (PMC9194872; doi:10.1016/j.jbc.2022.102030)
Supplement: Supplemental Figure Captions [file mmc5.pdf]

Figure S1. mTORC1 reactivation follows prolonged deprivation of leucine or arginine. mTORC1 associated signaling was assessed in MEFs that had been deprived of arginine or leucine alone **(A-C)** or all amino acids **(C,D)** for the indicated times, by immunoblotting endogenous proteins, as specified. p-ULK S757 / ULK, p-GRB10 S476/GRB10 immunoblot band intensity ratios are shown in **B**.

Figure S2. Analysis of Akt signaling in U2OS and 293T cells. Cell lysates of U2OS **(A)** or HEK 293T **(B)** that are shown in Figure 2 were reprobed with antibodies recognizing phosphorylation of Akt at T308 and S473. p-Akt S473 / pan-Akt and p-Akt T308 / pan-Akt immunoblot band intensity ratios are shown. Experiments were repeated at least twice with equivalent results.

Figure S3. Analysis of glutamine levels, and PI3K/Akt signaling in RagA/B-knockout cells. (A) MEFs were deprived of arginine or leucine for 0 minutes, 45 minutes, 3 hr, or 6 hr, and intracellular metabolite levels were analyzed via mass spectrometry. The graph shows relative glutamine levels from four separate experiments. Statistical data are presented as mean values ± standard deviation. ns = not significant ($P > 0.05$); one-way ANOVA with Tukey's post hoc test. **(B)** MEF cells were treated with either 4 mM (normal) or 8 mM (excess) glutamine (Gln) during the course of arginine starvation, whereafter mTORC1-related signaling was assessed by immunoblotting the indicated proteins. **(C-D)** HEK293A RagA/B double-knockout cells or control cells were deprived of arginine or leucine for the specified times and blotted for the indicated proteins. Experiments were repeated at least three times.

File S1. Uncropped immunoblots. Uncropped immunoblots of all western blots are shown in the order which they appeared.
